# Microbial lipases and their industrial applications: a comprehensive review

**DOI:** 10.1186/s12934-020-01428-8

**Published:** 2020-08-26

**Authors:** Prem Chandra, Enespa, Ranjan Singh, Pankaj Kumar Arora

**Affiliations:** 1 Food Microbiology & Toxicology, Department of Microbiology, School for Biomedical and Pharmaceutical Sciences, Babasaheb Bhimrao Ambedkar University (A Central) University, Lucknow, Uttar Pradesh 226025 India; 2 Department of Plant Pathology, School for Agriculture, SMPDC, University of Lucknow, Lucknow, 226007 U.P. India; 3 Department of Environmental Science, School for Environmental Science, Babasaheb Bhimrao Ambedkar University (A Central) University, Lucknow, U.P. India; 4 Department of Microbiology, School for Biomedical and Pharmaceutical Sciences, Babasaheb Bhimrao Ambedkar University (A Central) University, Lucknow, U.P. India

**Keywords:** Microbial lipase, Fatty acids, Triglycerides, Protein engineering, Biosensor, Food industry, *Candida antarctica* lipase B (CALB)

## Abstract

Lipases are very versatile enzymes, and produced the attention of the several industrial processes. Lipase can be achieved from several sources, animal, vegetable, and microbiological. The uses of microbial lipase market is estimated to be USD 425.0 Million in 2018 and it is projected to reach USD 590.2 Million by 2023, growing at a CAGR of 6.8% from 2018. Microbial lipases (EC 3.1.1.3) catalyze the hydrolysis of long chain triglycerides. The microbial origins of lipase enzymes are logically dynamic and proficient also have an extensive range of industrial uses with the manufacturing of altered molecules. The unique lipase (triacylglycerol acyl hydrolase) enzymes catalyzed the hydrolysis, esterification and alcoholysis reactions. Immobilization has made the use of microbial lipases accomplish its best performance and hence suitable for several reactions and need to enhance aroma to the immobilization processes. Immobilized enzymes depend on the immobilization technique and the carrier type. The choice of the carrier concerns usually the biocompatibility, chemical and thermal stability, and insolubility under reaction conditions, capability of easy rejuvenation and reusability, as well as cost proficiency. *Bacillus* spp., *Achromobacter* spp., *Alcaligenes* spp., *Arthrobacter* spp., *Pseudomonos* spp., of bacteria and *Penicillium* spp., *Fusarium* spp., *Aspergillus* spp., of fungi are screened large scale for lipase production. Lipases as multipurpose biological catalyst has given a favorable vision in meeting the needs for several industries such as biodiesel, foods and drinks, leather, textile, detergents, pharmaceuticals and medicals. This review represents a discussion on microbial sources of lipases, immobilization methods increased productivity at market profitability and reduce logistical liability on the environment and user.

## Introduction

The serine hydrolases are present in abundantly and known as lipase enzyme which belong to triacylglycerol ester hydrolase family (EC 3.1.1.3). They can catalyze the hydrolysis (and synthesis) of long-chain triglycerides to fatty acids, diacylglycerol, monoacylglycerol and glycerol known as carboxylesterases [1, 2]. Besides hydrolysis activity they display interesterification, esterification, aminolysis and alcoholysis activity which are contributed in wide range industries [3, 4]. Lipase synthesizes esters from glycerol and long-chain fatty acids in non-aqueous medium [5]. The microbial lipases are more valuable comparison to derive from plants or animals due to their variety of catalytic activities available, high yield production, and simplicity of genetic manipulation, absence of seasonal fluctuations, regular supply, more stability safer and more convenient and the growth rate of microorganisms very high in economically media [6, 7]. The bacterial isolates offer higher activities such as neutral or alkaline pH optima and the thermostability associated to yeasts [8]. Bacterial strains such as *Pseudomonas alcaligenes, P. aeruginosa*, *P. fragi*, *P. fluorescens* BJ‑10, *Bacillus subtilis, B. nealsonii* S2MT and some species of fungi are *Penicillium expansum*, *Trichoderma*, *Penicillium chrysogenum*; *Aspergillus niger* produces lipases in higher quantities [9–13]. The increasing awareness about animal health and quality of animal produce, and increasing consumption of enzyme-modified cheese (EMC) and enzyme-modified dairy ingredients (EMDI) the lipase market has been extensively increased [14, 15]. Due to the more benefits of microbial lipases over animal and plant lipases are also motivating the market growth. The request for microbial sources is projected to witness significant growth in the near future, due to their wide range of food processing applications [16, 17]. The microbial lipase market is projected to dominate due to cleaning agent segment through the forecast period [18]. The growth of industrial microbial lipases in the detergents industry is the innovative key factor to replacing harsh chlorine bleach with lipase and reduced the industrial as well as sewage pollution from fresh water [19, 20]. The microbial lipases in the form of powder is projected to dominate the microbial lipase markets due to its stability, easy to handle, and easier for packaging and its transportation preferred by the consumers [21, 22]. These are extensively applicable in several another industries such as dairy, food and beverage, animal feed, cleaning, biofuel, pharmaceuticals, textile cosmetic, perfumery, flavour industry, biocatalytic resolution, esters and amino acid derivatives, fine chemicals production, agrochemicals, biosensor, and bioremediation [23–25]. Additionally, altering in the dietary patterns have led to augmented the consumption of dairy products in the region; increasing in trepidations about superior hygiene, in consciousness of personal hygiene, contagious diseases, and bleaching household industrial surfaces [26]. The manufacturers who operate on a global level and the rising in implementation of lipase enzymes drive the demand for microbial lipases in the region [27, 28].

Between the 2015 and 2020, the market scope of lipase is expected to reach $590.5 Million by 2020 globally, at a CAGR of 6.5%. The Asia–Pacific was the largest market for lipase consumption in 2014 [29, 30]. And during the forecast period the Asia–Pacific market is estimated to grow at the highest CAGR. Moreover, the rising prospects in the developing markets such as India, China, and Brazil are expected to enhance the market scope of lipases over the forecast period. Novozymes A/S (Denmark), E. I. du Pont de Nemours and Company (Genencor) (U.S.), Koninklijke DSM N.V. (Netherlands), and Chr. Hansen Holdings A/S (Denmark) are the key industries reported for the consumption of lipases at worldwide (http://www.marketsandmarkets.com, 2020). Due to the specific properties such as enantioselectivity, regioselectivity and broad substrate specificity properties the lipase showing more interest between all the enzymes [31, 32]. This present review focused on discussing the sources of microorganisms, immobilization methods and their potential applications of lipases including commercially available.

## Historical background

Inside or outside the cells enzymes are proteins and have ability of catalyzing the various chemical and biochemical reactions. They are highly specific natural catalysts to the various types of substrates and operate under insignificant conditions of environmental factor such as temperature, pressure, pH, with high conversion rates [33, 34]. Lipase was first discovered in pancreatic juice as an enzyme by Claude Bernard in 1856, which hydrolysed unsolvable oil droplets and transformed them to soluble products [35]. After that the productions of lipase have been observed in the bacteria *Bacillus prodigiosus*, *B. pyocyaneus* and *B. fluorescens* in 1901, and in the current scenario *Serratia marcescens*, *Pseudomonas aeruginosa* and *Pseudomonas fluorescens* species of bacteria have been detected for the production of lipases on large scale [36]. Lipolase was the first commercial recombinant lipase industrialized from the fungus *Thermomycesl anugiwnosus* and expressed in *Aspergillus oryzae* in 1994 [37]. Traditionally, lipase has been achieved from the animal pancreas and was made applicable as digestive supplements in the form of crude or in purified grade. It has been extensively used as biocatalytic procedures for the synthesis of several novel chemical compounds [38–40].

### Definition of lipases

**Fig. 1:**
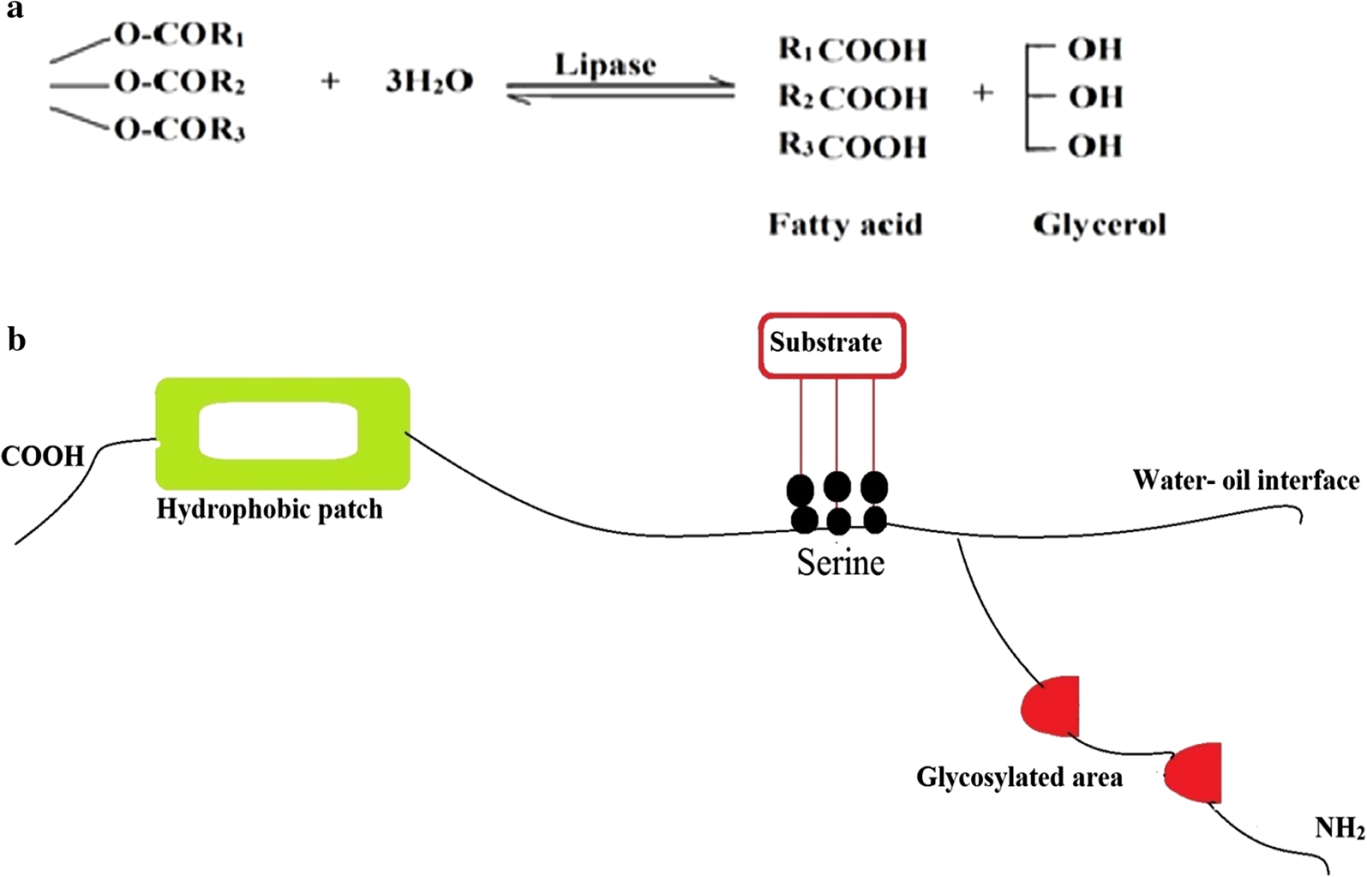
(**a**) Hydrolysis of triglyceride converts into glycerol and fatty acid. **b** Representation of a molecule of lipase with its features

Lipases (EC 3.1.1.3) are known as triacylglycerol acylhydrolase which acts on carboxylic ester bonds is the part of hydrolases family [41, 42]. They do not require any cofactor and belongs to the class of serine hydrolases [43]. Triglycerides hydrolyzed into diglycerides, monoglycerides, fatty acids, and glycerol by using the lipases naturally (Fig. 1a). The carboxylic esters bonds can be hydrolyzed by esterases in addition to lipases [44, 45].

The hydrolysis of ester bonds at the interface catalyzes by lipases between an unsolvable phase of substrate and aqueous phase where the enzymes keep on liquefied under natural conditions (Fig. 1b). However, *Pseudomonas aeruginosa*, *Candida anatarctica B*, and *Burkholderia glumae* possessed a lid but did not show interfacial activation [46, 47]. Esterification, transesterification, interesterification, acidolysis, alcoholysis, and aminolysis conversion reaction takes place by lipases [48, 49].

The presence of a lid and the interfacial activation are not the suitable criteria for to categorize a true lipase, carboxylesterase simply defined that catalyzes the hydrolysis and synthesis of long-chain acylglycerols [50].

### Properties and characteristics of lipases

The molecular weight of lipases is in the range of 19–60 kDa and reported to be monomeric protein. The position of the fatty acid in the glycerol backbone, chain length of the fatty acid, and its degree of unsaturation are the factors and the physical properties of lipases depend on it [51, 52]. The sensory and nutritive values of given triglyceride also affected by these features. Several lipases catalyze a number of useful reactions such as esterification due to their activeness in organic solvents [47, 53]. Lipases displayed pH dependent activities, generally at neutral pH 7.0 or up to pH 4.0 and 8.0 lipases are stable, *Chromobacterium viscosum, A. niger* and *Rhizophus* sp., produced extracellular lipases are active at acidic pH, and *P. nitroaeducens* produced alkaline lipase and active at pH 11.0 [54]. Under certain experimental conditions lipases have capability to reversing the reactions which leads to esterification and interesterification in the absence of water [55, 56]. For the expression of lipase activities the cofactors are not necessary but calcium is the divalent cation stimulates the activity [57, 58]. Co, Ni^2+^, Hg^2+^ and Sn^2+^ inhibited the lipase activities drastically and Zn^2+^, Mg^2+^, EDTA and SDS inhibited slightly. The half-life values determined temperature stability profiles of lipases and lower temperature shows more stability [59, 60]. According to the region-specificity lipases divided into two groups and revealed with acyl glycerol substrate. Without display of regiospecificity only fatty acids are discharged from all three positions of glycerols in the first group of lipases [61–63]. The fatty acids regio-specifically discharged from the 1, 3 positions of acylglycerols in the second group of lipase. Triacylglycerol hydrolysed by lipases and constructed 2-monoacylglycerol and free fatty acids 1, 2-(2, 3)-diacylglycerols. In *A. arrhizus*, *R. delemar, C. cylindracea* and *P. aeruginosa* the partial stereo-specificity have been detected in the hydrolysis of triacylglycerols [64–66]. These enzymes may be used to extract optically pure esters and alcohols due to these properties. At low water activity using the organic media offers an exceptional prospect over variation of the solvent [67]. So, varying the properties of the solvents an enzyme’s specificity may be transformed. Any solvent may utilize a substantial influence on the catalytic properties of an enzyme due to the possession of soft structures and delicate [68, 69].

### Kinetic model of lipolysis

**Fig. 2:**
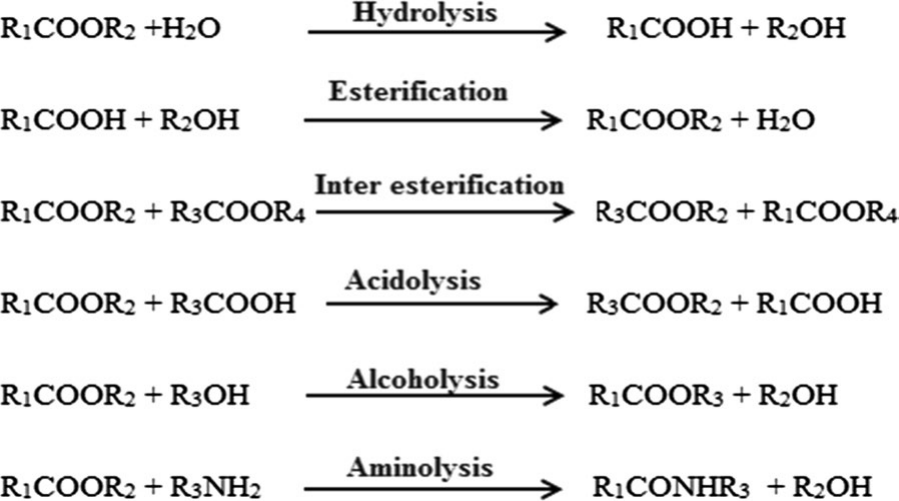
Lipase catalyzed different reactions

At the substrate/water interface lipolysis arises so the Michaelis–Menten model cannot be described it. In a homogeneous phase which is effective only for biocatalysis in which enzyme and substrate are soluble [70, 71]. At an interface to describe the kinetics of lipolysis simple models has been proposed and be made up of two consecutive equilibrium [72, 73]. The alterable adsorption of enzyme to the interface (E↔E*) happens in the first equilibrium phase, a single substrate molecule (S) binds by the adsorbed enzyme (E*) in the formation of (E*S) complex as a result in the second phase of equilibrium [74, 75]. For the enzyme–substrate complex to the Michaelis- Menten equilibrium this latter equilibrium is equivalent. Ending with the discharge of the products and renovation of the enzyme in the (E*) form, the subsequent catalytic steps take place once the (E*S) complex is formed [76, 77]. The adsorbed lipase in the vicinity of substrate concentration at the interface is at the surface concentration instead of volumetric concentration conventional in the atmosphere [78, 79]. The rejuvenated lipase remnant adsorbed to the interface and is only unrestricted after a number of catalytic cycles in this model (Fig. 2).

The activity of lipase is a utility of interfacial conformation: the enzyme can be denatured as well as triggered or neutralized and the interface is a suitable spot for restraining lipolysis. The directly interaction of lipase inhibitor with the enzyme and obstructs the activity of lipase. On the other hand, via the adsorption to the interphase or to the substrate molecules few compounds can postpone the lipolytic reaction [80–82].

Lipase inhibitors are grouped into two categories:

- Synthetic lipase inhibitors (including phosphonates, boronic acids and fats analogues) and
- Natural compounds (β-lactones and several botanical foodstuffs—plant extracts and metabolites, chiefly polyphenols, saponins as well as peptides and particular nutritive fibers). Lipases are essential enzymes for lipid absorption, so the absorption of fat or obesity controlled by the lipase inhibition. β- lactones including orlistat are the natural compounds, have the ability to inhibit the lipase activity [83, 84]. Over 80% of total dietary fats the pancreatic lipase is responsible for the hydrolysis. In several countries for the treatment of obesity orlistat is the registered drug [85].

### Lipase inhibitors from microbial sources

From microorganisms several metabolic products have potent pancreatic lipase (PL) inhibitory activity. The several bacterial, fungal and other marine species continued search of effective antiobesity agent screened to find new compounds with PL inhibitory activity [86, 87].

#### Lipstatin

The digestive activity of pancreatic lipases controls by the Lipstatin is a β-lactone molecule which also controls the absorption of fat in the small intestine. Lipstatin was first isolated from *Streptomyces toxytricini* is a precursor for tetrahydrolipstatin (also known as orlistat, Xenical, and Alli), the only FDA-approved antiobesity medication for long-term use is a very potent inhibitor of PL [88, 89]. Lipase inhibitory activity was lost on opening of β-lactone ring. The catalytic hydrogenation product of lipstatin is crystalline tetrahydrolipstatin and generally known as orlistat is currently on the market as an antiobesity agent [90, 91].

#### Panclicins

*Streptomyces* sp. NR 0619 produced Panclicins is another class of potent PL inhibitors. N-formylalanyloxy or N-formylglycyloxy substituent are two alkyl chains are found in Panclicins too contains b-lactone structures [80]. Panclicins A and B are alanine type while panclicins C, D and E are glycine type of compounds. The inhibitory activity was recognized to the amino acid moiety, alanine-containing compounds being two to three folds weaker than glycine-containing compounds [92].

#### Valilactone

Valilactone first isolated from *Streptomyces albolongus* MG147-CF2 strain from shaken culture and jar fermentation. Valilactone potently inhibited hog PL with an IC50 of 0.14 ng/ml. It also influenced inhibitory activity of esterase from hog liver with an IC50 value of 0.029 mg/ml [93].

#### Ebelactones

Ebelactone A and B are two ebelactones were isolated from the fermentation broth of Actinomycetes strain G7-Gl, closely related to *Streptomyces aburaviensis*. Both A and B revealed PL inhibitory activity with IC50 values of against hog PL are 3 ng/ml and 0.8 ng/ml, respectively [94].

#### Esterastin

Esterastin was isolated from actinomycetes *Streptomyces lavendulae* MD4-C1 strain from the fermentation broth. Competitively Esterastin introverted the hog pancreas lipase with IC50 value of 0.2 ng/ml [95].

#### Caulerpenyne

Caulerpenyne extracted and purified from an extract of *Caulerpa taxifolia* competitively introverted the activity of lipase with IC50 values of 2 mM and 13 mM, using creamed triolein and disseminated 4-methylumbelliferyl oleate as substrates, individually [96, 97]. The inhibitory activity of caulerpenyne was independent of substrate concentration suggesting direct interaction but dependent on the lipase concentration with the lipase protein, slightly than interacting with the substrate. Oral supervision of corn oil with caulerpenyne to rats demonstrated a reduced and hindered peak plasma triacylglycerol concentration, signifying its potential as a lipid absorption inhibitor [98, 99].

#### Vibralactone

Vibralactone secreted from *Boreostereum virens* microfungi is a scarce fused β-lactone-type metabolite, covalently but reversibly transforms the active site serine of the enzyme via acylation by the blactone. The IC50 of the vibralactone was resolute to be 0.4 mg/ml [94, 100].

#### Percyquinin

Percyquinin obtained from the cultures of Basidiomycetes *Stereum complicatum* ST 001837 [101], inhibited PL with an IC50 of 2 mm, is another β-lactone metabolite. In one study on β-lactone class of compounds, the stereochemistry (2S, 3S) of the β-lactone ring was found to impart specificity for the PL, while (2R, 3R) stereochemistry was accountable for inhibition of HMG-CoA synthase [80].

## Sources for microbial lipases

Microbial lipases found universal in nature and are commercially substantial due to the low manufacturing cost superior stability and more availability than animal and plant lipases [102]. Naturally or recombinant microbial lipases are generally used in diverse bioengineering applications [103]. A wide diversity of microbial resources provides by nature, microbes have more adaptation abilities and inhospitable atmospheres like Dead Sea, Antarctica, Alkaline lakes, Hot springs, volcanic vents and contaminated soils, which provides extraordinary potential for the lipases production with specific features [104, 105]. An enormous spin-off with esteem to the enantioselectivity hydrolysis and the formation of carboxyl esters has produced ready availability. The marine microfloras have more capabilities for the formation of enzymes and proteins active compounds. Mostly lipase fashioned extracellularly secretion from fungi and bacteria [106, 107].

In numerous biocatalytic procedures *Candida antarctica* lipase B (CALB) is the most habitually used enzyme and have a more amount of patents. *Candida rugosa* lipase (CRL) is another scientifically significant lipase from the yeast, which is a mixture of different isoforms and is commercially accessible and this grounding is known as “Generally Recognized As Safe” (GRAS) and used in the food industry [108]. PLA1s and PLA2s from *Fusarium oxysporum, T. lanuginosus, A. niger* and *Trichoderma reesei* between the yeast and fungal phospholipases are used in the degumming of vegetable oils and commercialized. While mostly used in the food industry are PLA1s, PLA2s and PLBs extracted from *A. oryzae* and *A. niger* [103, 109]. Due to their high transphosphatidylation and hydrolytic activities PLDs isolated from *Actinomycete* strains are commercially available and used in several industrialized procedures [110]. Mostly the bacterial genera for the production of lipases and phospholipases have been reconnoitered are *Pseudomonas*, *Bacillus* and *Streptomyces*, followed by *Burkholderia*, *Chromobacterium*, *Achromobacter*, *Alcaligenes* and *Arthrobacter* [111]. Some lipases producing microorganisms reveal new sources and applications of industrial enzymes as shown in Table 1.

**Table 1:** Microbial source of Lipase and their industrial application

Microbial Sources	Applications	References
Fungal species		
*Fusarium solani* NFCCL 4084	Halophilic lipase for biodiesel production	[470]
*Yarrowia lipolytica*	Degrades very efficiently hydrophobic and unusual substrates such as n-alkanes, oils, fats, and fatty acids as low-cost carbon sources	[670]
*Aspergillus oryzae*	Saturated fatty acids synthesized, faster cheese ripening, flavour customized cheese	[671]
*Rhizomucor javanicus (meih)*	Non-hydrogenated solid fats	[672]
*Rhizomucor miehei*	Cocoa-butter equivalents	[673]
*Geotrichum candidum* and *C. antarctica*	Through biocatalytic processes preparation of chiral intermediates which synthesized the pharmaceutical compounds related to the elimination of bad cholesterol for the treatment of the Alzheimer’s disease	[674]
*Candida antarctica*	Oils and fats enriched, removal of size lubricants, denim finishing	[675]
*Candida rugosa*	Human Milk fat substitute	[676]
*Candida lipolytica*	Cheese ripening, Fatty acid production	[670]
*Penicillium camembertii*	Production of glycerolglycolipids	[672]
	Synthesis of saturated triacyl glycerides	[677, 678]
*Trichoderma lanuginosus*	Produced a lipase containing detergent ‘LipoPrime^®^’	[16]
*Penicillium roquefortii*	Production of characteristic flavor of blue cheese in dairy products	[679].
*Aspergillus niger*	Faster cheese ripening, flavor customized cheese, Dough stability and conditioning	[680]
*Meyerozyma guilliermondii*	Promising feed lipase using cheese whey	[681]
*A. niger* GZUF36	Potential of the enzyme in the synthesis of functional oils	[526]
*Aspergillus flavus*	Fat stain elimination; Synthesis of pharmaceuticals, polymers, biodiesels, biosurfactants	[682]
*Candida antarctica*	Pitch control in paper and pulp industry, Polycondensation, ring opening polymerization of lactones, carbonates in polymer	[674]
*Rhizomucor meihei*	As a biocatalyst in personal care products such as skin and sun-tan creams, bath oils etc	[683]
*Rhizomucor meihei*	Surfactants for baking industry, dairy products, Noodles	[684]
*Rhizomucor miehei*	Oils and fats enriched, cocoa butter substitutes, synthesis of bioactive molecules	[685]
*Candida tropicalis, Aspergillus oryzae*	Degradation of crude oil hydrocarbons	[686]
*Penicillium abeanum*	Use for docosahexaenoic acid enrichment of tuna oil	[687]
*Rhizopus nodosus*	Leather processing and dehairing and fat removal	[688]
*Candida rugosa*	Activated sludge treatment, aerobic waste treatment	[689]
*P. chrysogenum*	Food industry waste treatment	[690]
*Rhizomucor meihei*	Surfactants for baking industry, Dairy products, Noodles	[684]
*P. chrysogenum*	Food industry waste treatment	[690]
*Thermomyces lanuginose*	Non-hydrogenated solid fats	[691]
*M. miehei*	Used as aroma and fragrance in the food, beverage, and pharmaceutical industries	[692]
*C. parapsilosis*	Hydroxamic acids (food additive)	[534]
*M. miehei, C. antarctica*	Synthesis of short chain flavour thio-ester in solvent free medium	[643]
*M. miehei, Rhizopus arrhizus*	Production of flavour esters	[693]
Bacterial species		
*Achromobacter* sp. HEGN 014, *Virgibacillus pantothenticus* HEGN 114	Treatment of oily wastewater	[694]
*Pseudomonas mendocina*	Dishwashing/laundry Removal of fat strain	[622]
*Acinetobacter radioresistens; Bacillus* sp. FH5	Used in detergent industry	[695]
*Staphylococcus pasteuri*	Using in oil degradation	[696]
*P. fluorescens*	Enantioselective transesterification of a racemate (*R*,*S*)-4-methyl-1-heptyn-4-en-3-ol, a component of the insecticide S-2852	[697]
*Staphylococcus warneri* and *S. xylosus*	The production of flavour esters	[693]
*Bacillus* sp.	Used in leather processing	[698]
*Brevundimonas* sp. QPT-2	Involved in enantioselective degradation of AOPP herbicides	[699]
*Micrococcus* sp.	Commonly used detergents, enhance the removal of oily stains from various types of fabrics	[448]
*Bacillus cereus* HSS	Waste water treatment	[626]
*Marinobacter lipolyticus*	Organic Solvent-Tolerant Lipolytic enzyme	[700]
*Haloarcula* sp. G41	Organic solvent-tolerant lipase for biodiesel production	[701]
*Bacillus subtilis*	Baking industry for bread making	[702]
*Geobacillus stearothermophilus*	Enhanced stability in methanol	[449]
*Pseudomonas aeruginosa* HFE733	Biodegradation of oil and organics (determination as chemical oxygen demand (COD), biodegradation of food wastewater from restaurants	[703]
*Pseudomonas* sp.	Food processing and oil manufacture	[704]
*Natronococcus* sp.	Application in biocatalysis	[701]
*P. alcaligenes* M-1	Alkaline lipases, able to removing fatty stains when used in a washing machine	[705]
*Pseudomonas plantarii*	Solvay Enzyme Products, Applicable for is a nonionic and/or anionic detergent formulation	[706]
*Chromobacterium viscosum*	Detergent formulations containing alkaline lipase used in laundry detergent “Top”	[707]
*Acinetobacter* sp.	Degrading 60–65% of the fatty material in the waste water management	[708]
*Bacillus thermocatenulatus*	Used in medical industry	[641]
*Lactobacillus casei, Lactobacillus paracasei, Lactobacillus rhamnosus,* and *Lactobacillus plantarum.*	Cheese Industry for improvement of flavor	[709]
*Penicillium roquefortii*	Cheese Industry for cheese ripening	[710]
*Staphylococcus warneri, S. xylosus*	Production of flavour esters	[711]
*Pseudomonas cepacia*	Biodiesel fuel production	[712]
*Pseudomonas* sp.	Formation of (−)-15-deoxyspergualin 23) in drug industry as antitumor antibiotic and immunosuppressive agent	[713]

### Bacterial lipases

Lipase has been detected initially in 1901, *B. prodigiosus* and *B. fluorescens*, presently *Serratia marcescens* and *P. fluorescens* observed today’s best lipase producing bacteria subsequently [112–115]. The glycoproteins and lipoproteins are bacterial lipases. In most of the bacteria the enzyme production is affected by the certain polysaccharides have been observed [116–118]. Some bacterial lipases are thermo-stable and most of the bacterial lipases are reported as constitutive and nonspecific in their substrate specificity [119, 120]. *Achromobacter* sp., *Alcaligenes* sp., *Arthrobacter* sp., *Pseudomonas* sp., *Staphylococcus* sp. and *Chromobacterium* sp. have been exploited for the manufacturing of lipases between the bacteria [121].

### Fungal lipases

Since 1950′s fungal lipases have been studied, due to their affluence in thermal and pH stability, substrate specificity, and activity in organic solvents and downstream processing these lipases have been exploited [122]. The contemporary period machinery favors the procedure of batch fermentation and low cost extraction methods so the fungal lipases have assistances over bacteria. Major filamentous genera of fungi included are *Rhizopus, Aspergillus, Penicillium, Mucor*, *Ashbya, Geotrichum, Beauveria*, *Humicola, Rhizomucor, Fusarium, Acremonium, Alternaria, Eurotrium* and *Ophiostoma* for the production of lipases [123, 124]. Other species such as *Candida rugosa*, *Candida antarctica*, *T. lanuginosus*, *Rhizomucor miehei, Pseudomonas, Mucor* and *Geotrichum*. *Colletotrichum gloesporioides* produced 27,700 U/l of lipase are the most productive strain identified from the Brazilian savanna soil by using enrichment culture techniques [125, 126]. *A. niger, C. rugosa, H. lanuginosa, M. miehei, R. arrhizus, R. delemar, R. japonicus, R. niveus* and *R. oryzae* are the principal manufacturers of these commercial lipases [127–129].

## Purification of lipases

To get consistency of lipase from a large number of bacteria and fungi various novel purification technologies are available [130]. Generally, several steps are contains for the purification of lipases contingent upon the purity estimated for food application. The extracellular microbial lipases from the culture broth eliminated by the centrifugation or filtration in the fermentation process and cells are became freed [131, 132]. The ammonium sulphate precipitation, ultrafiltration or extraction with organic solvents is concentrated the cell-free culture broth [133]. The gel filtration and affinity chromatography like several combination of numerous chromatographic approaches purified about of the 80% using precipitation steps, and then 60% ammonium sulphate and 35% ethanol. A homogenous product produces is the final step of gel filtration [134].

The novel purification machineries such as the (i) membrane separation procedures, (ii) immuno purification, (iii) hydrophobic interaction chromatography using epoxy-activated spacer arm as a ligand and polyethylene glycol restrained on Sepharose, (iv) polyvinyl alcohol polymers as column chromatography stationary phases, and (v) aqueous two phase systems are frequently engaged after these pre-purification steps [135, 136]. The enzyme recovery and fold purification outcomes are found acceptable using of hydrophobic interaction chromatography [137, 138]. An acid resilient lipase has been filtered from crude profitable arrangements by size exclusion on Bio-gel-p-100 and ion exchange on Mono-Q., From *A. niger* fungi. Using the chromatography on hydroxyapatite, octyl-Sepharose and sephacryl S-200 the lipase was purified to homogeneity from *R. japonicus* NR400 [139].

## Substrates for lipase

A chiral alcohol moiety possesses by the glycerides which is the natural substrate for lipases. The lipases were mostly valuable for the resolution or asymmetrization of esters bearing a chiral alcohol moiety was assumed [140–143].

## Methods for lipase assay

Due to the wide substrate specificity of lipases a number of assay protocols are engaged for lipase assay. At the lipid water interface the determination of lipase activity is the analytical of free lipase [144]. Using various physiochemical approaches the determination activities can be carried as with all reactions catalyzed by enzymes and observing the vanishing of the substrate or by the product release [145]. For the determining of the hydrolytic activity several methods are presented such as Titrimetry, Spectroscopy (Photometry, Fluorimetry and Infrared, Chromatography, Radio activity, Interfacial tensiometry, Turbidimetry, Conductimetry, Immunochemistry, and Microscopy [146, 147]. The triacylglycerol hydrolysis reaction catalyzed by lipases generally can be written as:

$$ \begin{aligned} {\text{Triacylglycerols}} \to {\text{Diacylglycerols}} + {\text{Free}}\,{\text{fatty}}\,{\text{acids}} \to {\text{Monoacylglycerols}} + {\text{Free}}\,{\text{fatty}}\,{\text{acids}} \hfill \\ \to {\text{Glycerols}} + {\text{Free}}\,{\text{fatty}}\,{\text{acids}} \hfill \\ \end{aligned} $$

The activity of lipases can be examined by the monitoring of release of either free fatty acids or glycerol from triacylglycerols or fatty acid ester displays by this reaction [148]. The titrimetery assay using olive oil as a substrate is the mostly used lipase assay protocol due to its simplicity, correctness and reproducibility [149, 150]. Another spectrophotometric assay based on techniques which purify the colour to fatty acids releasing after the hydrolysis of triacylglycerols [151, 152]. The release of 1 μmole of free fatty acid from combined olive oil or triolein or tributyrin per minute at specified temperature and pH values which relates a lipase activity unit. The units of lipolytic activity per microgram of extra cellular protein expressed the Specific activity of lipases [153].

## Immobilization of lipases

Recyclability, enzyme stability and activity of expensive lipases improve due to the immobilization process. It can easily control the process of enzymatic reaction purity of the products and for its reusability feature [36]. Multi-fold benefits such as increase in thermal and ionic stability are applicable using immobilized lipases which upturns its proficiency. When the enzyme is immobilized it is easier to control reaction parameters like flow rate and substrates convenience [154, 155]. For immobilization include large surface area, low cost, reusability, good chemical, mechanical and thermal stability, and insolubility the desirable characteristics of solid supports used. According to the interface among the enzyme and support the enzyme immobilization approaches can be classified like physical and chemical procedures [156]. The interactions among the enzymes and support are by weaker bonds like hydrogen bonds, Van derWalls exchanges, which create these interactions adjustable in the physical method. For the interface among the enzyme and support are stronger by covalent bonds the procedure created irrecoverable in chemical methods [157, 158].

### Physical methods

#### Adsorption

In the physical approaches of immobilization adsorption procedure, the enzymes immobilized by Van der Waals bonds, hydrophobic interactions, hydrogen bonds, and ionic bonds [159]. On the surface of the support the enzyme becomes adsorbed (bound), and the substrates used mostly for this procedure are cation and anion exchange resins, activated carbon, silica gel, alumina, controlled pore glass, ceramics, natural materials like cellulose and agarose, additionally to specific industrialized deposits [160]. The procedure of absorption is modest, low cost and takes two phases to comprehensive it; in the first dispersion of the enzyme happens through the conveyors surface, and then conveyor adsorption. Adsorption is controlled by the diffusion for lipase since the support binding is quicker than its diffusion [161]. The immobilization occurs naturally and the process is executed under slight conditions, subsequently, without affecting its catalytic activity there is no change or slightly change in the structure of enzyme [36].

#### Encapsulation and entrapment

In fiber or gel of synthetic polymeric or natural sustenance these approaches include captivity or casing the enzymes. In the one step these methods provides easy contact between enzymes and substrate along with increased mechanical stability are effective and low cost [162]. In the second step, encapsulation and entrapment decrease mass transferal of substrate to enzyme because of small matrix pores size, but the large apertures to permit leaking enzymes from the carriers [36]. Enzyme deactivation during the procedure of immobilization is another disadvantage, during use abrasion support material, and small loading capacity. So, this difficulty may be resolved by cross-linking mediator addition [160].

#### Confinement

Confinement also known as imprisonment in the immobilization of enzymes the unification of the enzyme is the part of a reactive mixture to be polymerized, generally the porous matrix is formed around the biocatalyst to be immobilized in this procedure [163]. The polymer matrix encircles the enzyme confining it in its structure and the substrates and produces diffuse through the support as the polymerization profits, while the enzymes linger immobilized within the provision [158, 164]. The immobilization technique in captivity is simple and fast comparatively being one of the approaches of relaxed use in insignificant procedures, and very appropriate in the manufacture of biosensors, because there is limited chance of conformational changes of the enzymatic structure, thus its catalytic action permitting, besides the prospect by less cost arrays [165, 166]. However, the effort in monitoring the size of the pores of the support can principal to the leaching of enzymes and also restrictions in developing the diffusion of substrates and yields inside the support, besides the striving in upgrade are the main problem in immobilization [167]. The enzyme activity is highly dependent on the type of immobilization demonstration. These outcomes designated that the immobilized lipase not only showed good recovery of activity but also significant stability, better reuse, and flexibility to use than free lipase by entrapment and adsorption [168].

### Chemical methods

#### Covalent binding

The lipases are immobilized to support of chemical bonds in the covalent variety in this immobilization process. These bonds arise from chemical reactions like glutamic acid residues, lysine, cysteine, and aspartic acid among these carrier ingredients and the side chain amino acids of the enzymes and hydroxylamine, carboxylic, imidazole, and phenolics functional groups are found for the development of covalent bonds additionally [169]. Covalent immobilization delivers enzymatic stability and high add-on of the lipases to support, ensuring rigidity in its structure because it is a strong chemical bond [170]. The structure of the enzyme can maintain by this rigidity and unaffected against denaturing agents like organic solvents, heat, extreme pH, and others. Though, the active site of the enzyme changes by the covalent bond producing its inactivation [171]. For this to materialize through activation reactions the surface of the support is modified as long as the improvement of more forceful functional groups for interface with the group’s enzyme. Consequently, the selection of support containing a more concentration of reactive groups to permit the enzyme-support binding is very imperative [172].

#### Cross-linking

The crosslinking used to increase the stability of enzymes is a technique of enzymatic immobilization, for the enzyme to bind which does not involve support. Using a reagent called crosslinking agent or crosslinker the immobilization process is carried out, which form intramolecular and intermolecular crosslinks with specific groups of amino acids present on the surface of the solubilized enzyme consequently formed crosslinked enzymes [160, 173]. To protect the enzyme from the external environment is the main function of crosslinking agents. Enzymes obtained by crystallization, atomization, and aggregation leads by crosslinking approaches [174]. The immobilization of the enzyme arises subsequent in the development of Cross-Linked (soluble) Enzyme (CLE), Cross-Linked Enzyme Crystals (CLEC), Crosslinked Enzyme Aggregates (CLEA), and Cross-Linked Spraydrying Enzyme (CSDE) when these enzymes are placed in a medium containing a cross-linking agent [175, 176]. Due to the elimination of solid support; besides being an adaptable process are highly catalyzed enzymatic activities, high stability, and low cost of production is the main advantage of cross-linking process, it is possible to get more healthy and stable enzymes for industrial uses [177, 178].

### Cross-linked enzyme

Enzymatic cross-linking to be recognized was the first process, but the other differences of the techniqes looked are enzymatic crosslinked enzyme crystals, crosslinked enzymatic aggregates, and atomized crosslinked enzymes. CLE that arises by crosslinking among the dissolved enzymes and the crosslinker agent is an immobilization technique [179].

#### Cross-linked enzyme crystals

A crosslinking agent is added into the solution containing the crystallized enzyme using an immobilization technique known as CLECs. The conformational structure of the enzyme, as well as its catalytic activity may modify this procedure [180]. The immobilization process of the crystallized enzyme monitors from its addition to the crosslinking agent, generally which is a two-dimensional solid surface; the enzyme stabilization arises that means forming a three-dimensional structure of intermolecular and intramolecular crosslinks that perform as a barrier evading its solubilization in the medium [181], to alterations in the reaction medium production it more resistant, temperature, pH, permitting storage for long periods (up to years), and consenting its recycle easing the parting of the medium; due to the high enzyme concentration moreover all these benefits, when compared to immobilized or soluble enzymes CLECs present higher volume catalytic activity [164, 182]. Though, CLEC have needed of that the enzyme be crystallized, it is necessary that it is highly purified but to crystallize the enzyme, which is a very costly process, assembling the immobilization procedure sophisticated and expensive [183].

#### Cross-linked enzyme aggregates

The CLE procedure has their catalytic activity reduced if the enzymes immobilized, because they are solubilize in the solution, discussed previously. To achieve the crystallized enzyme in the CLEC procedure, to be immobilized which must be purified highly, the procedure formed very costly and complex [184, 185]. So, the CLEAs appeared as a substitute to the procedures defined in the literature already. The precipitating agents like salts, acids, organic solvents addition by the precipitated enzymes using the protein precipitation procedure [179, 186], and in the mixture holding the enzyme without distressing its active three-dimensional structure. As a result, the lacking need for it to have the topmost clarity and obtaining the desired enzyme, and reducing the cost of immobilization and time [187].

#### Cross-linked spray-dried enzyme

With a crosslinking agent blending spray-dried enzymes the cross-linked spray-dried enzymes (CSDEs) are produced. In this method, a polymer (carrier particles) a solution/suspension containing the enzyme is fed into a spray dryer; to escape deterioration of enzymes due to disclosure to high temperatures these particles are used during drying [184]. To a medium containing the crosslinking agent the enzymes are added so that crosslinking occurs after drying. To control various parameters it is technically possible, like as particle size of the enzyme, due to the deactivation of the enzyme that occurs during the spray drying the application may be limited [188].

## Industrial applications of lipases

### Application in food industry

#### Lipase in dairy industry

For the hydrolysis of milk fat, to modify the fatty acid chain lengths and to boost the flavour of cheeses lipases are widely used in the dairy industry [189, 190]. Currently, it is also applicable in the speeding up the ripening of cheese and lipolysis of fat, butter and cream. By the action of lipases on milk fat various products particularly soft cheeses with specific flavour characteristics generated with free fatty acids [191, 192]. For the production of cheese from *M. miehei, A. niger, A. oryzae* etc. the engineered industry developed a whole range of microbial lipases [156, 193]. Using the individual microbial lipases or their mixtures for the preparations of a good quality range of cheeses produced [194]. At raised temperature in the presence of enzyme when cheese is incubated Enzyme Modified Cheese (EMC) is manufactured and in order to harvest a concentrated flavour using lipase catalysis [195]. In comparison to normal cheese in EMC the concentration of fat is 10 times higher and used as an ingredient in other products like dips, sauces, soups and snack [196, 197]. Acetoacetate, β-keto acids, flavour esters, methyl ketones and lactones flavour ingredients are synthesized due to the free fatty acids by the initiating of simple chemical reactions [198, 199]. In vitamin A and E esters the lipase catalyzed hydrolysis and alcoholysis of ester bonds. To the oxidation prone vitamins A and E the Supercritical Fluid Extraction (SFE) technique are used for the research of immobilized *C. antarctica* [200–202]. For the determination of vitamins D2/D3, K53 and β-carotene in milk powder and infant formulations the SFE technology should be applicable. Several cheese types, such as cheddar, provolone and ras cheeses the gastric lipases are applied to hasten the ripening and improvement of flavour [203]. The rate of fatty acid deliverance augments after the addition of lipase which also hastens the growth of flavour [204, 205]. The liberation of fatty acids significantly increased with the adding of calf lipase and aggregates the ripening temperature (from 7° to 53 °C) [206, 207]. Liberated fatty acid profiles of the enhanced procedure were undistinguishable to the control and the entire amounts of short-chain liberated fatty acids (C4 to C6) were significant for the improvement of cheddar cheese flavour during maturing revealed in the observations [208, 209]. Remains the lipase to be active after maturing and can cause the improvement of strong rancid flavour his is the disadvantage. A highly soluble proteins and free fatty acids and displayed better flavour within 3 months of ripening in the cheddar cheese industrialization when a cock-tail of fungal protease and lipase were used [210, 211]. During the ripening of a high level of enzyme may result in too much enzymatic reaction communicate an undesired specific and decrease the productivity [212, 213]. For faster cheese ripening decreases bitterness and losses in productivity the liposome technology adopted [214]. By cell lysis the bacterial intracellular enzymes are unrestricted and subsidize to flavour through lipolysis and other enzymatic actions [215]. Cell free extracts microcapsules in milk fat can be added to takeout milk clotting. With intact capsules formed cheeses contains more enzymatic end products significantly than the acquired by enzyme addition directly [216, 217]. By encapsulating in a high melting fraction of fat the capsule stability can be upgraded. In cheese the inherent milk lipase made from unpasteurized milk which affects the substantial lipolytic action [218, 219]. In Blue-vein and Camembert cheeses are lipolytic and produce lipases using the culture and secondary microflora such as *P. roqueforti* and *P. camembertii*, respectively [220, 221]. Paramesan, Provolone, and Romano are Italian cheese to intensify their flavour after adding the lipases generally [222, 223]. There is a steady increase in the concentration of progressive fatty acids and total soluble nitrogen during nitrogen [224, 225]. Triggering the development of cheese flavour lipases releases the fatty acids from triglycerides. In dairy foods the overview of conjugated linoleic acid (CLA) has been complete possible by the immobilization of lipases [226]. Both lipases and proteases accelerate ripening of cheeses individually as well as a “cocktail”. As such the enzymes may be added or encapsulated [227, 228]. A series of enzymatic reactions proceeded very gradually during the cheese ripening, transforming the fresh, automatically worked curd to the anticipated final ripe cheese texture and flavour [229]. Lipases, proteases and lactase enzymes hydrolyze lipids, proteins and lactose, respectively in order to elevate the level of flavour moieties and/or flavour mainframes [230, 231].

#### Lipase in fat and oil industry

In food processing manufacturing the oil and fats amendment is one of the prime areas which demands economically green technologies and it is very significant constituents of foods [232, 233]. Changing the location of fatty acid chains lipases permit us to amend the assets of lipids in the glycerides and interchanging one or other of these with new ones [234, 235]. Relatively economical and less appropriate lipid can be improved to a higher value fat in this way. The hydrolysis, esterification and inter esterification of oils and fats catalyzed by the fat [236, 237]. Esterification and inter esterification are used to get value added products between the lipolytic transformation of oils and fats like specialty fats and partial glycerides using the positional and fatty acid detailed lipases, and have superior industrial prospective than fatty acid production in bulk through hydrolysis [238, 239]. For fat and oil hydrolysis an immobilized lipase membrane reactor assembled which produced products and that involve less downstream processing so reduced the overall cost of processing [240, 241]. Highly selective microbial phospholipases is a recently industrialized environmental friendly procedure for the removal of phospholipids in vegetable oils (de-gumming) [242, 243].

To production of a food grade, cost effective, immobilized 1, 3-regioselective (lipozyme TL 1 M) lipase using granulation to immobilize lipases, targeted for the production of frying fats and for the inter esterification of commodity oils reductions and lard components [244, 245]. To produce modified acylglycerols lipases catalyzed interesterification of fats and oils it cannot be acquired by predictable chemical interesterification [246, 247]. For the esterification of functionalized phenols and production of lipophilic antioxidants using immobilized lipases from *C. antarctica* (CAL-B), *C. cylindracea* Ay30, *H. lanuginosa, Pseudomonas* sp. and *G. candidum* to be used in sunflower oil [248]. Lipases used in the pure form, in the immobilized form or in the cell bound form on the hydrolysis of fats and oil observed in the many studies [249, 250]. In 2002, Australia New Zealand Food Authority (ANZFA) the use of triacylglycerol lipase achieved from hereditarily modified *A. oryzae* as a processing aid in the oils and fats productiveness for oil de-gumming, and in the food industry to progress emulsifying possessions was scientifically accepted [251]. Based on the granulation of silica a new procedure for immobilizing lipases has intensely shortened the development and let down the procedure cost. For the manufacture of commodity fats and oils with no content of trans-fatty acids such inventive methods are now extensively employed [252, 253].

A continuous packed bed rector for the design and operation was established for the interesterification of soybean oil having 22.7% oleoyl and 54.3% linoleoyl moieties as molar acyl in hexane consuming an immobilized Sn-1, 3-specific lipase (Lipozyme IM) from *M. miehei* with oleic acid [254, 255]. The loss of catalytic activity of Lipozyme IM in soybean oil reduced the rate of change in oleoyl and linoleoyl moiety arrangements in soybean oil decreased. The lipase catalyzed acidolysis of soybean oil with oleic acid to increase oleic acid content in an organic solvent [256, 257]. The degumming step can be conceded out with a phospholipase in the physical refining of vegetable oils. By the introduction of a microbial phospholipase (Lecitase Novo) the economy of enzymatic degumming has been improved expressively [258, 259]. Glycerolysis of commercial oils and fats catalyzed using Novozym 435 (*C*. *antarctica* lipase) to form monoacylglycerides (MGs) was examined using a tetra ammonium established ionic liquid as the reaction medium [260]. Nearly 100% alteration of triglycerides in this ionic liquid produced a 90% of monoglycerides were accomplished which were significantly higher as associated to the productivity in normal solvents [261]. For the retailoring of vegetable oils microbial lipases may be exploited. The nutritionally important structured triacylglycerols such as coco butter substitutes, low caloric triacylglycerols, and PUFA and oleic oil-enriched oils may be upgraded using cheap oils [262, 263]. By using directed interesterification normally the fat and oil alterations are carried out chemically and known as non-specific and energy intensive. Lipase intervened alterations occupy a noticeable place in oil industry for tailoring structured-lipids since enzymatic alterations are specific and can be carried out at moderate reaction conditions [264, 265].

A structured lipid (SL) from natural vegetable oils synthesized and contains EFAs and natural antioxidants [266]. To produce oils and fats containing nutritionally important polyunsaturated fatty acids (PUFAs) enzymatic interesterification can be used known as eicospentaenoic and docosahexaenoic acids. The interesterification of triglycerides using immobilized lipase was not commercially viable due to the high cost and the processing [267, 268].

#### Lipase RM and lipase TL for cocoa butter analogues

The vegetable oils origins such as palm, rapeseed, canola, and sunflower used by food producers regulates the physical properties of fats and oils since every oil has several types of fatty acids in the 1, 2, 3 positions of triacylglycerides a diverse scattering [269, 270]. Exploiting the microbial lipase that are 1, 3 regio-specific [6], used in the production of cocoa butter-type triacylglycerols principally and catalyzed interesterification using lipase [271]. Using 1, 3 regio-specific lipases the interesterification has been used to enrich low-cost fats like palm-oil fractions into 1, (3) palmitoyl, 2-oleoyl, 3 (1) stearoylglycerol and 1(3) stearoyl, 2-oleoyl, 3(1) stearoylglycerol, which have enormous presentation as confection fats [272, 273]. Crystallization possessions as well as melting features are formed in chocolate using 30% cocoa butter. Cocoa butter has a tendency to be very expensive so an additional source from fat assortments was industrialized which requires an original mixing of palm mid fraction and stearate ester; monitored by desiccation and enzymatic lipase reactions [274, 275]. The distillation and solvent fractionation are essential for the compulsory product formation in further processing. In marketable manufacturing of cocoa butter this procedure has been used comprehensively equivalent by Loders Croklaan of the Uniliver Group in Wormerver, Netherlands [276]. Due to steric deterrent lipase is sn-1, 3 specific characteristically do not interchange acyl groups at the 2 position are produced from *Mucor miehei, Rhizopus arhizus, Aspergillus niger*, and *Thermomyces lanuginosus,* though some intramolecular transesterification of diacylglycerol intermediates can occur over prolonged reaction times [277, 278]. The production of a cocoa butter equivalent the enzymatic transesterification was initially assessed that activate the sn-1, 3 specificity of a diversity of fungal lipases [279, 280].

#### Lipases used in cosmetics and personal care products

The cosmetic market globally share may surpass USD 680 billion by 2024, using related to hair care, skin care, perfume, personal hygiene, oral products. For the advanced cosmetic merchandises may drive the growth of industry. Lack of regulatory policies may hinder supply dynamics and put pressure on lipase market price trend [281, 282]. The manufacturing of isopropyl myristate, isopropyl palmitate and 2- ethylhexylpalmitate for the application as a palliative in particular maintenance products like dermal and sun-tan ointments, bath oils etc. prepared by Unichem International (Spain) [283]. An essential fragrance component in the perfume industry rose oxide formulated from several microbial sources with lipases and Transesterification of 3, 7-dimethyl- 4, 7- octadien-1-ol. The immobilized lipase of *Rhizomucor meihei* was used as a biocatalyst [284, 285]. In place of the conventional acid catalyst the used enzyme provides needful lowest downstream refining and plentiful advanced value claimed by company. In personal care products wax esters (esters of fatty acids and fatty alcohols) have related uses and are also being enzymatically manufactured (Croda Universal Ltd.), and in a batch bioreactor the company uses *C. cylindracea* lipase [286]. Normally, the production cost is marginally higher than that of the conventional techniques according to the manufacturer, and the upgraded quality of final product justified cost. In makeups and pharmaceuticals like skin care products the abundant commercial potential of retinoids (Vitamin A and their derivatives) are found. In the catalytic reaction of immobilized lipase the water-soluble retinol derivatives were prepared [287–289]. And the lipases also used for the hair stressing provisions, and as a constituent of topical antiobese emulsions or as oral administration. They are also used for the cleaning, moderating, aroma, and coloring in personal care of cosmetic sector [290, 291]. The lipases show activities in surfactants and in aroma production and used in cosmetics and perfumeries also. In the presence of lipase a patent Nippon Oil and Fats obtained from for the preparation of propylene glycerol mono fatty acid ester and also used as emulsifier and a pearling agent in cosmetics and foods [292, 293]. Novozym 435, derived from *Candida antartica* is a nonspecific lipase and determined for the enzymatic combination of isopropyl myristate most suitably [294, 295].

#### Lipases used in tea processing

Tea is the most popular beverage and manufactured from the *Camellia sinensis* L. in the world. But the process of manufacturing is different for each one such as unfermented is green tea, semi-fermented is oolong tea, well fermented tea is known as black tea [296]. Tea that is commercially available is prepared from the bud of the plant and apical two leaves of *Camellia sinensis* (L). To complete tea depends on the effect of oxidative and hydrolytic enzymes present endogenously in the green leaf the renovation of fresh tea leaf. The tea leaves processing can be done either by the orthodox process or the Cut-Tear-Curl (CTC) process. The orthodox method, though very extravagant, produces tea of high quality that is light and aromatic. The main feature of the CTC process is that it is much simpler but results in teas with more cuppage and lesser aroma. For these motives, the CTC teas are more economical than the orthodox ones. If the flavour of CTC teas can be enhanced to the level of orthodox teas, it would be a favourable cooperation between superiority and economy [297]. Enzymatic breakdown of membrane lipids initiate the development of volatile products during the manufacturing of black tea with specific flavour properties accentuate the significance of lipid in flavour improvement [298, 299]. The quality of black tea is depending upon dryness, mechanical breaking and enzymatic fermentation to which tea leaves are exposed. The level of polyunsaturated fatty acids detected by the reduction in total lipid content which enhanced by *Rhizomucor miehei* lipase [300, 301].

#### Lipase used as biosensors in food industry

Two important part of biosensor with their unique properties are combined as physico-chemical transducer is used as measurable signal and the second compartment is biological origin for the providing specific analysis [302, 303]. One of the parts of biological origin entitled is the Lipase. And a common substrate tributyrin designated for the origins of various lipases. Several bacteria *Bacillus subtilis* and *Chromobacterium viscosum* and fungus *Rhizomucor miehei, R. oryzae, Fusarium solani* hydrolases tributyrin to dibutyrin and butyrate are mentioned for the lipases [304, 305].

For the quantitative determination of triacylglycerol the immobilized lipases are used as biosensor due their accuracy and efficiency. Lipases are essential in the food industry specifically in fats and oils, soft drinks, drug industries, beverages, and also in medical diagnosis [306]. Using the lipase enzymes as biosensor in the analytically and quantifically methods the triacylglycerol breakdowns into the glycerol. For the determination of organophosphorous pesticides using the lipase hydrolysis a surface acoustic wave impedance biosensor developed [307, 308]. And it is also used for the analysis of Dichlorvos insecticide residues in vegetables. Blending with glucose oxidase the lipases may be immobilized onto pH/oxygen electrodes, and functions as lipid biosensors also used for the analysis of triglycerides and blood cholesterol samples [309, 310]. Presently, the chromatographic and spectroscopic methods are inadequate for the quantification and the determination of pesticidal residues in water and food grains. The estimation and the detection of the triglycerides is a clinically significant parameter and which is correlated to the disorder of heart related problems [311–313]. Another biosensor industrialized for the analysis of methyl-parathion and tributyrin was potentiometric biosensor based on *C. rugosa* lipase. The purified lipase *C. rugosa* was immobilized on glass electrode and cross-linked with glutaraldehyde [314, 315]. The compound 4-nitrophenyl laurate hydrolysed into 4-nitrophenol and laurate, 4-nitrophenyl oleate into 4-nitrophenol and oleate, 4-nitrophenol palmitate into 4-nitrophenol and palmitate, 4-nitrophenyl propionate into 4-nitrophenol and propionate, α-naphthyl acetate into α-naphthol and acetate, methyl acetate into methanol and acetate, methyl butyrate into methanol and butyrate, methyl laurate into methanol and laurate, methyl palmitate into methanol and palmitate, methyl propionate into methanol and propionate, and methyl stearate to methanol and stearate by *Micrococcus* sp. was verified [316–318]. The overview of Electrochemical and Optical assays-based on lipase biosensor are specified in following Table 2.

**Table 2:** Electrochemical assays at lipase based Biosensor

Source of used lipase	Analyte	Principle of lipase use in assay	Detection limit	References
Electrochemical assays at lipase based biosensor				
*Candida rugosa* (Fungi)	Methyl parathion (p-nitrophenyl pesticides)	On a glass pH electrode lipase was mobilized and transformed which reduced the pH; methyl-paraoxon inhibit reaction	93 μmol/l	[714]
*Burkholderia cepacia* Lipase (Bacterium)	Methyl parathion, (p-nitrophenyl)	Lipase was immobilized on zeolitic nanoparticles and then into chitosan on a glassy carbon electrode, pesticides like methyl parathion were hydrolyzed to p-nitrophenyl that was electrochemically oxidized in the next ste	0.1–38 µM/l	[715]
*Candida rugosa* (Fungi)	Diazinon	Lipase converted diazinon to diethyl phosphorothioic acid and 2-isopropyl-4-methyl-6- hydroxypyrimidine. which caused a change in the impedance of the medium	10 nmol/l (fungal lipase)	[306]
*Candida rugosa* (Fungi)	Chlorfenvinphos, Malathion	Lipase converted p- nitrophenyl acetate to p- nitrophenol and acetic acid, p- nitrophenol was oxidized and a current at 0.024 V was recorded, analyzed inhibited lipase and stopped the reaction.	84.5 µmol/l for chlorfenvinphos and 282 µmol/l for malathion	[716]
Optical assays-based on lipase biosensor				
*Candida antarctica,* *Yarrowia lipolytica* and fungus	Lipase itself	p-nitrophenyl butyrate hydrolysis to butyric acid and p-nitrophenol, coloration caused by p-nitrophenol was measured	0.05 U/ml	[717]
*Candida antarctica,* *Mucor miehei,* *Thermomyces lanuginosus* (Fungus) and bacteria *Pseudomonas* *cepacia* and *P. fluorescens*	Lipase itself	Butyryl 4-methyl umbelliferone (Bu-4-Mu) and methanol in tert-butanol were trans-esterified in the presence of lipase, production of 4-methylumbelliferone was measured fluorometre	Not available	[718]

### Nanotechnology in enzyme biosensors

In biosensor planning a significant role played by the nanotechnology, less than 100 nm smaller dimensions which involves in the study of manipulation, creation, and use of materials, devices. Incorporating enzymes with nanomaterials the electrochemical biosensors are new ingredients with synergistic possessions initiated from the apparatuses of the hybrid combinations [319–321]. A new generation of bioelectronics devices with high sensitivity and stability has an excellent scenario based on nanotechnology biosensors. To achieve direct wiring of enzymes to electrode surface using nanoscale materials this promotes electrochemical reaction, commanding nanobarcode for biomaterials, and signal amplifying of biorecognition event. Carbon nanotubes (CNT) and gold are regularly used nanomaterial for enzyme biosensors [322, 323]. Gold showed more catalytic ability for several organic reactions. So, to catalyze biochemical reactions to design biosensors metal nanoparticles have been used. Additionally, in the reaction medium the nanoparticles perform as predictable identical catalysts, but after the reaction can be easily recovered [324–326]. CNT rolled up into a nanoscale are graphite sheets, having diameters range among fractions of nanometers and tens of nanometers and lengths up to numerous centimeters with both their ends generally covered by fullerene-like arrangements [327, 328].

#### Immobilized enzymes in the food industry

In several methods the enzyme is immobilized such as adsorption, entrapment, and covalent binding on several supports. Immobilization creates thermostability of the enzyme and prevents the loss of enzyme activity. The technical circumstantial behind enzyme immobilization for superior catalysis is multifarious [181]. In addition to the easiness of handling and the use of two main targetted profits is (a) easy split-up of enzyme from the products, (b) reuse of the enzyme [160]. Easy split-up of the enzyme from the product simpilifies enzyme use and supports a dependable and effectual reaction tools. The reuse of enzymes delivers cost benefits which are often an important requirement for establishing an enzyme catalysed procedure in the first place. The immobilized enzyme arrangements possessions are administered by both properties the enzyme and the carrier material [329].

#### Lipase CalB for vitamin C esters

**Fig. 3:**
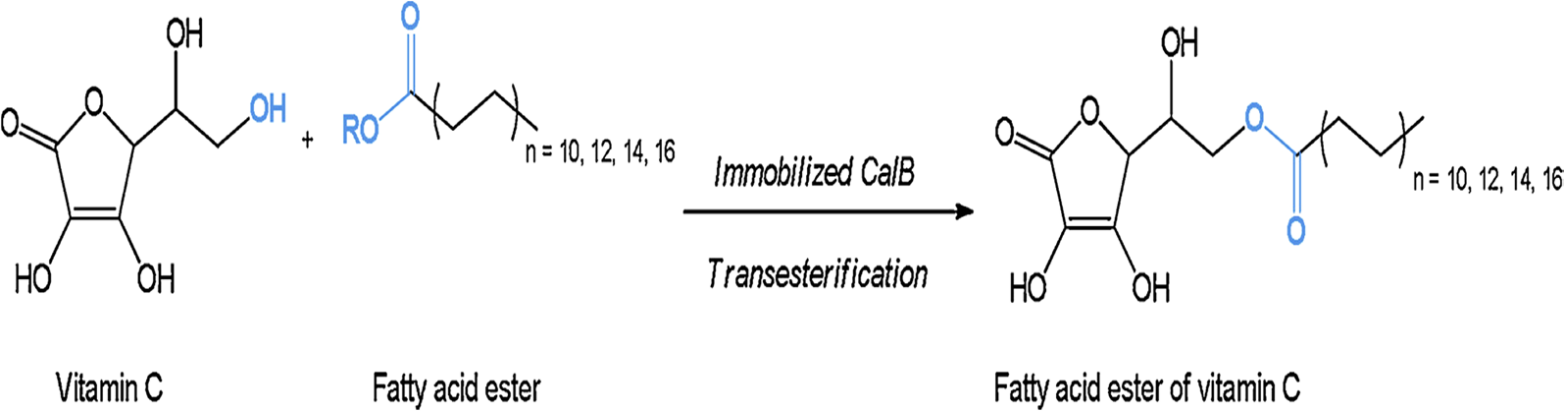
Manufacture of vitamin C fatty acid ester by transesterification catalyzed by immobilized CalB [344]

L-Ascorbic acid (Vitamin C) acting as a free radical scavenger react with oxygen consequently removing it in a closed system is the foremost water-soluble usual antioxidant [330, 331]. Vitamin C is insoluble in oils and fats compared to esters of L-ascorbic acid are soluble in fats with long-chain fatty acids (E-304) are active as antioxidants in foods rich in lipids [332, 333]. Ascorbic acid reacting with sulphuric acid produced ascorbyl palmitate and stearate monitored by re-esterification with consistent fatty acid, and successively distilled by re-crystallization. The need for tedious merchandise isolation, less productions due to non-regioselectivity reactions and the use of strong acids has some shortcomings due to this chemical process [334]. From *Candida antarctica* (CalB) the immobilized lipase B described by biocatalytic methods as biocatalyst and free fatty acids or activated esters such as acyl donors (Fig. 3). The conversion of biocatalytic can do levels of approximately 95% alteration contingent on operational temperature, the productivity of the side product (water) removal, and length of the fatty acid [335–337].

Comparatively with the current chemical procedures the enzymatic synthesis offers selected benefits like lower reaction temperatures, cleaner product and decreased downstream processing [68, 338]. The production of ascorbyl esters mostly is still achieved by chemical synthesis so this biocatalyzed procedure is stagnant in its original improvement period [339, 340]. The enzymatic procedure and the high costs of the restrained enzymes linked to the chemical catalysts due to the long reaction time requirement [341, 342].

#### Lipase in human milk fat substitutes

Several lipids such as oleic (30–35%), palmitic (20–30%), linoleic (7–14%) and stearic acids (5.7–8%) are found in Human milk fat (HMF) [343]. Palmitic acid is the major saturated fatty acid in HMF contrasting in vegetable oils and in cow’s milk fat commonly esterified at the sn-2 position of the TAGs while the unsaturated fatty acids are at the external positions [343, 344]. HMF fatty acid profile has a vital effect in infants on its digestibility and intestinal absorption. With free fatty acids (FFA) from different sources the sn-1, 3 lipase-catalyzed acidolysis of tripalmitin, butterfat, palm oil, palm stearin or lard (rich in palmitic acid in sn-2 position) the Human Milk Fat Substitutes (HMFS) have been obtained [343, 345]. Through the acidolysis between lard and soybean fatty acids catalyzed by the sn-1,3 selective lipase from *Rhizomucor miehei* (Lipozyme^®^ RM) using IOI Loders Croklaan, by biocatalytic processes the commercial Betapol^®^ product is industrialized [245, 346].

#### Lipase used in egg processing

**Fig. 4:**
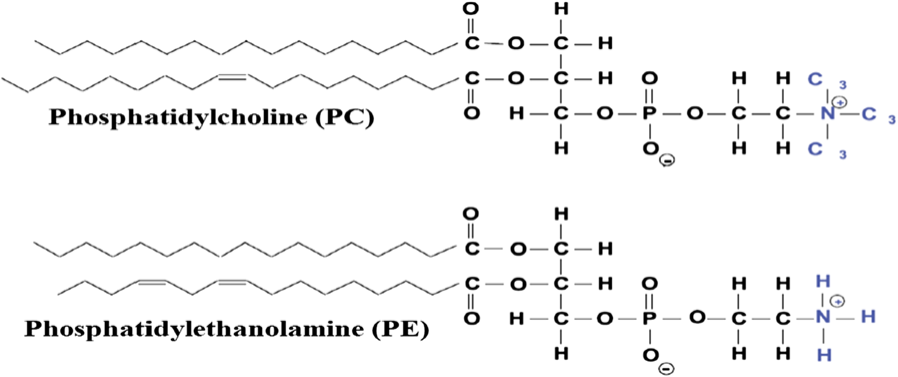
The emulsion stability increased of egg yolk phospholipids into lyso-phospholipids by the enzymatic conversion [669]

A variety of properties such as foaming, gelation, emulsifying in batters and mayonnaise and enhanced texture of baked goods eggs provides practical constituents to the food industry [347, 348]. The emulsifying properties of the egg lipids improved by the lipases for the better performance and the addition rate of lesser egg yolk in managed food recipes, like dressings and mayonnaise-like products, so the egg lipids are accountable for the emulsifying possessions [349, 350]. In the Russian and East European countries is concentrated one-third of the market for emulsified dressings. Nestlé, Kraft and Unilever are the global players and highly industrialized market [351]. The emulsified dressings of egg yolk are assessed at 3 millions of metric tons per year globally for the production in their process and consumed approximately 150 000 metric tons per year [352, 353]. Egg yolk is composed of 50% water, 32% lipids and 16% protein a complex oil–water emulsion, in which 80% lipids is phoshatidylcholine (PC) and approximately, 1/3 of the lipids are phospholipids [354, 355]. And it also contains phosphatidylethanolamine (Fig. 4).

#### Lipase in bakery products, confectionery and cheese flavourings

For the hydrolysis of milk fat in the dairy industry lipases are expansively used. In current uses of lipases are the flavour improvements of cheeses, the cheese ripening hastening, the cheese like products manufacturing, and the butterfat, cream lipolysis [356]. By the action of lipases on milk fat provides many dairy products from free fatty acids with their specific flavour features particularly the soft cheeses [357]. The short chain (mainly C4 and C6) fatty acids released primarily with the addition of lipases which leads the improvement of a sharp, tasty flavour, while the announcement of medium chain (C12, C14) fatty acids have a habit of to report a soapy taste to the product [220, 358]. Additionally, the free fatty acids participate in simple chemical reactions and the transformed by the microbial inhabitants of the cheese [359, 360]. The acetoacetate, beta-keto acids, methyl ketones, flavour esters and lactones flavour gradient are manufactured by their initiations [361]. In the production of enzyme modified cheeses (EMC) lipases plays a significant role [362]. In the presence of enzymes EMC incubated at raised temperature in appropriate to harvest an intense flavour for the use as an essential in new merchandises such as dips, sauces, dressings, soups, snacks, etc. *Mucor meihei* (Piccnate, Gist-Brocades; Palatase M, Novo Nordisk), *A. niger* and *A. oryzae* (Palatase A, Novo Nordisk; Lipase AP, Amano; Flavour AGE, Chr. Hansen) and several others a whole range of microbial lipase preparations has been industrialized for the cheese engineering industry [363, 364]. In coffee whiteners the enhancement of flavour to yield the creamy flavour, and buttery texture of toffees and caramel lipases has been used. Lipase enzyme produced from *Pencillium roqueforti* developed blue cheese flavour [365, 366]. *C. antarctica* lipase fraction B (CAL-B) can be involved as a strong biocatalyst in the esterification reactions due to the high transformations accomplished in the synthesis of short-chain flavour esters in an organic solvent, though this enzyme showed diffident enantioselectivity with chiral short-chain carboxylic acids [367, 368]. In the existence of beef extract/butter oil and lipases the fermentation of *Candida utilis* monitored by spray drying manufactured yeast which had a beefy/blue cheese like flavour [369, 370]. In the manufacturing of better-flavored alcoholic beverages this improved yeast can be used. *A. niger, R. oryzae, C. cylindracea* are produced lipases and used in bakery products [371], which expedites bakeries to extend shelf-life of breads, increase and control the non-enzymatic browning, increase loiter volume and progress the fragment structure [372, 373].

#### Lipase used in wine

The color, taste and aromatic properties of wine contributed a complex mixture of thousands of compounds [374]. Much attention has been received on wine aroma in current years and several sensory compounds recognized [375]. The ethyl esters have received pronounced attention due to its great influence on taste between these compounds [376, 377], and the ethyl acetate ester is most collective compound represent in wines. Though, the other esters like ethyl decanoate, ethyl 2-methyl-propionate, ethyl 3-methyl-propionate, ethyl 3-methylbutanoate, ethyl cinnamate, methyl-butyl acetate, 2-phenyl–ethyl acetate and hexyl acetate, 2-ethyl hidroxpropionato, diethyl butanediato, ethyl butanoate, ethyl hexanoate, octanoate [378, 379]. During the aging process of the beverage those formed enzymatically between an alcohol and an acid formed by chemical esterification are classified in two groups [380, 381]. Stability in the presence of ethanol, sodium metabisulfate, malic, tartaric, citric and lactic acid and high activity on pH 5–7 together with the specific properties of esterases and lipases used for the production of ethyl acetate, ethyl butanoate, ethyl hexanoate and ethyl octanotate [382, 383]. *Escherichia coli* BL21 genetically improved by insertion of gene encoding the lipase/esterase enzyme consequential from *Lactobacillus plantarum* WCFS estimated for the characteristics of lipase/esterase production [384]. The microorganisms produced enzymes and presented a high potential for the application in wine production procedure and showed high activity at low pH and stability in the presence of ethanol, sodium metabisulfite and tartaric, lactic and citric acids [385, 386]. During the processing of papaya wine the effect of sequential inoculation of yeasts, *Williopsis saturnus* var. *mrakii* NCYC2251 and *Saccharomyces cerevisiae* var. bayanus R2 evaluated on ester production [387].

#### Lipase used in dietetics

There are increasing the demand for low caloric fats and fat replacers due to the risk associated with high fat intake so the awareness increased of consumers in current scenario [388]. Todays the fatty acid contains majority of reduced caloric fats and fat substitutes available, and these are not present in edible oils and fats naturally but match the chemistry and functions of the natural fats [389, 390]. But the disadvantage of such products is the deficiency of nutritionally significant essential fatty acids (EFA) [391]. The structured triglycerols formed a positional analysis and showed a proliferation for the primary positions paralleled to the secondary positions in preference of the lipase action [392, 393]. In the Sn-2 position the targeted structured triglycerols with palmitoyl moieties and in the Sn-1, 3 positions of medium chain acyl moieties should be beneficial for infant nutrient and clinical in food formulation as well as parental sustenance uses [394, 395]. Lipase isolated from *R. miehei* was used as the biocatalyst for the acidolysis are commercially immobilized Sn-1, 3-specific lipase, Lipozyme RM IM. The incorporation level increased with reaction time for both oleic and stearic acids [396, 397]. The SLs produced have potential use in infant formulae and stated for the collaboration among industry and academia for increasing the successful commercialization of enzymatic processes [398]. Infant formula production with more absorbs TAGs with modification of vegetable oils [233]. The Poly Unsaturated Fatty Acids (PUFAs) and Medium Chain Fatty Acids (MCFA) in the same positions and amounts composed amounts as those found in human milk [399, 400]. The structured lipids (SLs) having palmitic, oleic, stearic and linoleic acids, similar to human milk fat (HMF), were manufactured by enzymatic acidolysis between tripalmitin, hazelnut oil fatty acids and stearic acid [401, 402]. *Staphylococcus epidermidis* is the gram-positive bacterium the lipase treated formulas incapacitated it [312, 403]. The lipid fraction of infant formulations is not only a source of nutrients they also worked as the antiviral and antibacterial activity incubation with lipases shows in current studies [404, 405].

#### Lipase in meat and fish industry

To remove excess fat in the meat manufacturing and fish industry to produce the lean meat the lipase are also used. It is also used to enhance its flavour for the fermentation of meat products [406], and to expand the superiority of fermented sausages. For the hydrolysis of fish oil and aggregate the unsaturated fatty acid (n-3 PUFA) the microbial lipases are also used [407]. A fish processing by-products diversity contains growth factors contribution decent prospective as culture media, as displayed by the highest level of lipase activity formed by several microbial strains. With the use of fish wastes for microbial lipase manufacture associated with a major concern is the presence of lipids [408]. Using *Staphylococcus epidermidis* CMST Pi2 a defatted fish meat improvement allowable a maximum lipase manufactureing [409]. So, that there is a convinced need for improving the microbial lipase manufactureing taking into contemplation of several factors particularly the composition of fish waste and microbial strain nutrient necessities [410].

In the beginning at the times of scarcity the dry-curing was used as a meat preservation process; due to the spread use of refrigeration technology while today it has lost their importance. Though, the process has been reformed and amended in order to obtain a palatable and gorgeous meat product [411, 412]. Adipose tissue lipids and muscles are also ingredient to intense lipolysis, manufacturing free fatty acids by the lipases action that, in another stage, is renovated to volatiles as a consequence of oxidation [413]. Dry-cured hams are strongly affected the sensory profiles of by these enzymatic reactions [414]. Additionally, the muscle enzymes activity level is depend on the properties of raw ham significantly, such as crossbreeding age and the environmental factor of process like temperature, time, water activity, redox potential, and salt content [415, 416]. Therefore, the muscle enzyme system controls lipases and proteases generally, are vital for the normalization of the processing and/or improvement of flavor eminence of dry-cured ham [417–419].

#### Other applications of lipases

In the formation of biopolymers the bacterial lipases are also applied. To produce solvent tolerant lipase used for the synthesis of ethyl butyrate ester in non-aqueous environment the *B. multivorans* V2 was observed [420, 421]. In the presence of *Pseudomonas* lipase by alcoholysis of cod liver oil the Omega-3 polyunsaturated fatty acids are prepared. *Pseudomonas* sp. produced lipase and can be used for the production of isopulegol compounds has fragrance like citrus and spearmint flavor occurred by b-pinene [422].

### Agriculture applications

#### CalB lipase as herbicide for dimethenamids-P

**Fig. 5:**
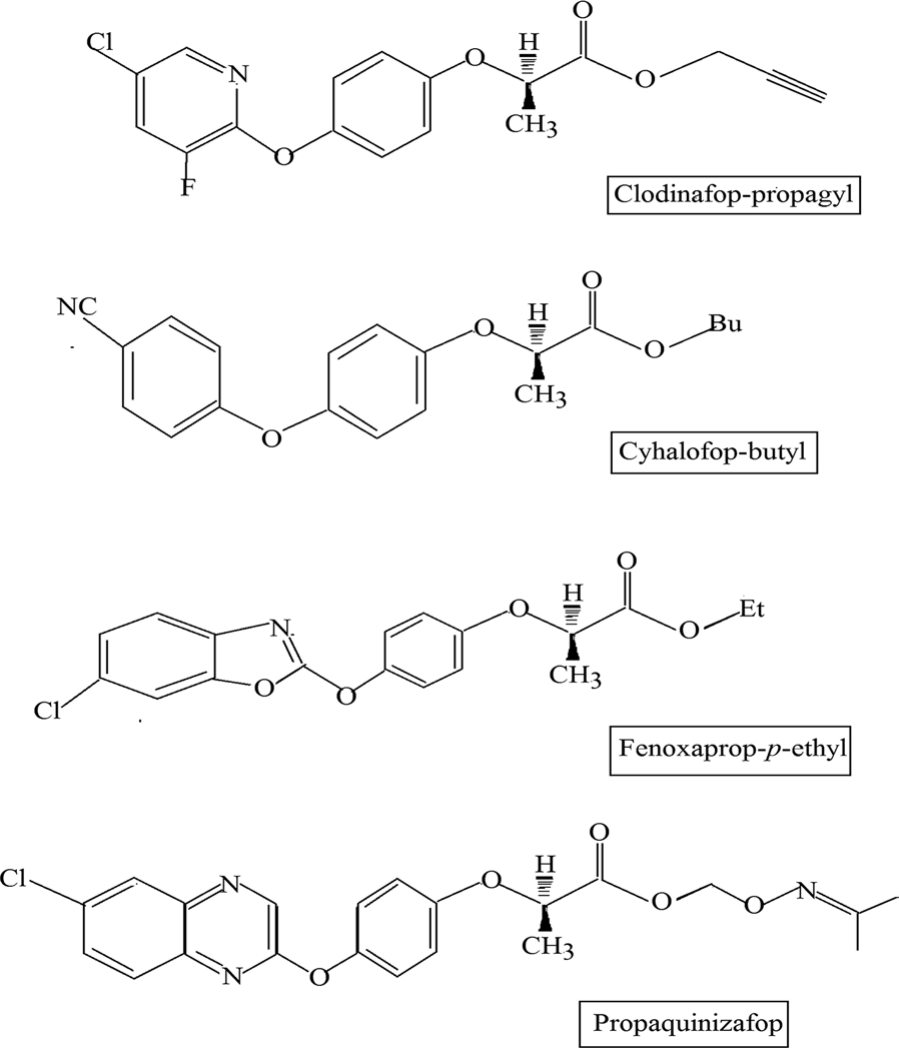
Phenoxypropionate herbicides examples with chiral center and R-configuration

By the enzyme inhibition of fatty acid biosynthesis a selective group of herbicides which are active against targeted plant species [423]. A number of active the aryloxy-substituted phenoxypropionate with a chiral center possess a common chemical structure (Fig. 5). A chief part of the herbicidal activity resides in only one of the enantiomers was identified similarly in pharmacological molecules.compounds within this group.

At position 2 the R-configuration is the most active so there has been substantial power from enterprises to improve the economical paths to manufacturing particular enantiomers. Instead of a racemate using a single enantiomer reduced the cost of producers and for the formers and also reduced the environmental influence [424, 425]. So, the biocatalysis substantiated to be an exceptional apparatus to succeed such enantioselectivity. The compounds Metolachlor and Dimethenamid were industrialized as a racemic mixture initially. Subsequent their innovation, it was recognized that a foremost quantity of the herbicidal movement be present in individually one enantiomers [426, 427].

**Fig. 6:**
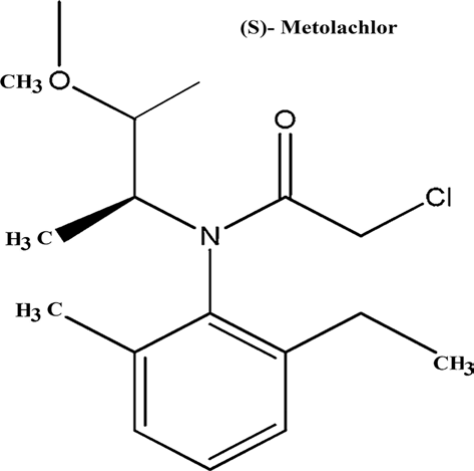
(S)-Metolachlor chemical structure

**Fig. 7:**
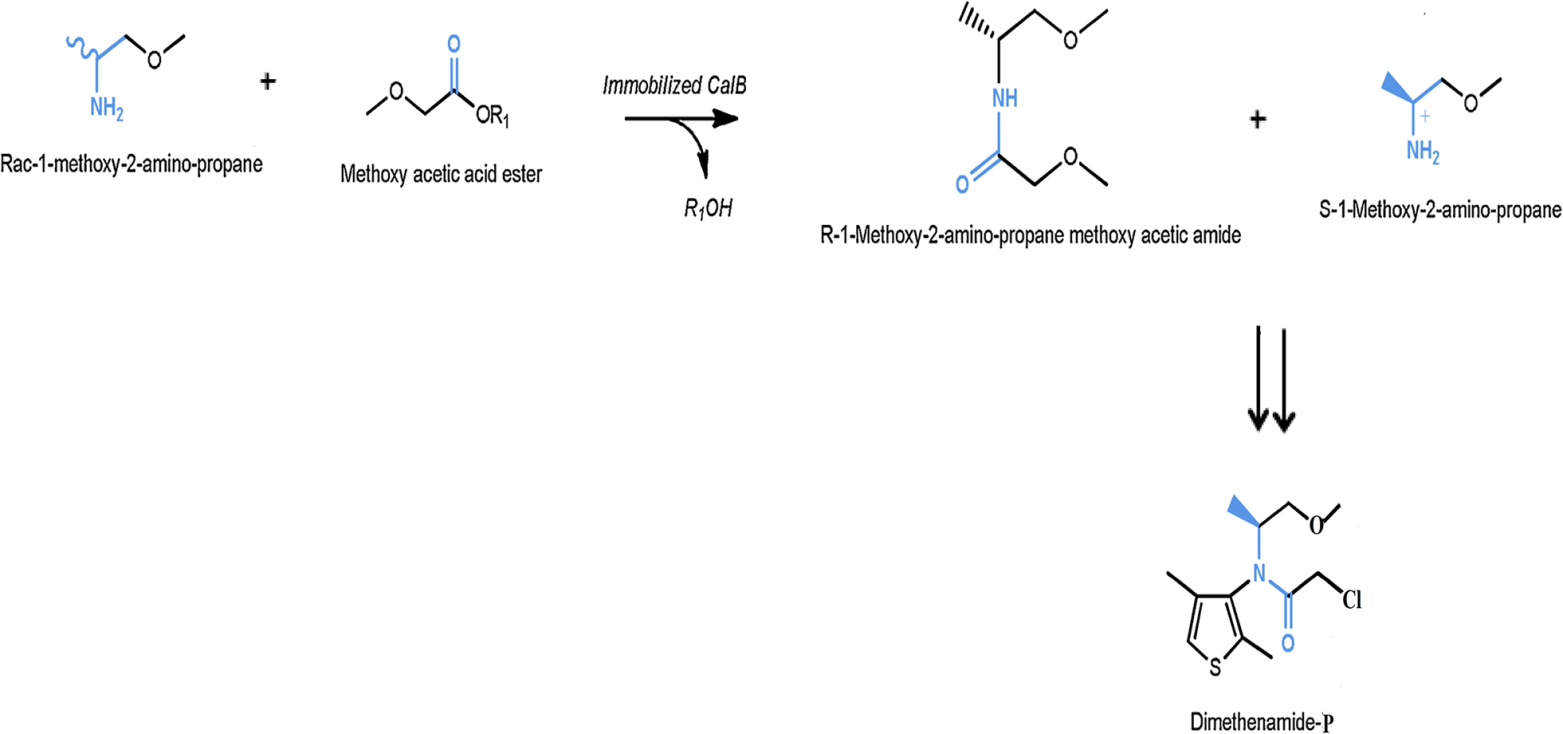
Dimethenamide-P formation using immobilized CalB by enantioselective transamination

The dynamic ingredient in Dual Magnum^®^ (for the use of maize a chief grass herbicides), in the case of (S)-Metolachlor (Fig. 6), an imine hydrogenation with the enantiomerically pure ligand “xyliphos”, consumed in the manufacturing process of chemical and iridium catalyst system catalyzed it [428, 429]. These process of dimethenamid industrialized by Sandoz 1996, sold to BASF, and the route to the single enantiomer Dimethenamid-P developed successfully (Fig. 7), a selective acylation of (S)-1-methoxy-2-aminopropane with ethyl 2- methoxyacetate involves or immobilized lipase CalB catalyzed a longer chain ester [344].

The molecule chirality lies in the 1-methoxy-2-aminopropane part of the structure in the case of dimethenamid [430]. Without addition of organic solvent using neat reagents the reaction takes place at 20–60 °C and accomplishes transformations of > 60% with (S)-amine ee > 99% using immobilized CalB (Novozym 435 or L2 from Chirazyme), however the undesirable (R)- amide is reprocessed further [431]. Due to the extraction or distillation process the undesirable (R)-amide and alcohol by-product can be upgraded the desired (S)-amine. To obtain the Dimethenamid-P the improved (S) amine is further reacted [432]. By the using immobilized lipase this process was further simplified and in the process can be separated easily and for racemic resolution cycles further used [356, 433]. By BASF and others in form of (R)-amide the process was also advanced allowing the reuse the unwanted enantiomer. BASF sold Dimethenamid-P as commercial product under the brand name of Outlook™. Small-seeded broadleaf weeds such as water hemp, pigweed and nightshade and other grasses the growth was retarded by this product [311, 434].

### Applications of lipases in environment cleaning

#### Lipase in waste water or effluent treatment

Both aerobic and anaerobic approaches lipases are comprehensively applied in the treatment of wastewater. One of the most important aerobic treatment devices formed known as activated sludge process, the thin layers of fats from the surface of aerated tanks to permit oxygen transport constantly removes includes maintenance of biomass [435]. Lipases used from *C. rugosa, Pseudomonas, Bacillus, Acinetobacter* etc. [436] can be easily digested the impurities of skimmed fat-rich. Lipases are widely useful in the industrial wastewaters treatment such as, food waste, dairy waste, and grease from wool, manure and waste water from oil mills employing the anaerobic processes [437, 438]. The waste water effluents of food processing, tannery, automobile industries, and restaurant and fast-food outlets treated from lipase producing bacteria and seeding them. The regular performance of anaerobic digesters assisted using lipases [311, 439, 440]. In the wastewater treatment plants the treatment of fats using enzymatic approaches mainly triglycerides can be hydrolyzed up to 90% filth and further improved using immobilizing lipase synthesizing bacteria [441]. The genetic engineering approaches of microorganisms in the treatment of effluents also successfully applied [442].

#### Lipase in bioremediation

To decontaminate samples from oil spills, oil-wet soils, industrial wastes and wastewater tinged with lipids the process employed is known as bioremediation [443]. Without the prior treatment of the effluent which enters into the natural environment may be hazardous. *Staphylococcus pasteurii* COM-4A, *Bacillus subtilis* COM-B6, *Arthrobacter* sp. are the lipase-producing organisms to curb the contaminants effectively have been reported [444, 445]. The species of *Pseudomonas* assisted as accessible apparatuses for microbial remediation such as *P*. *aeruginosa* have been particularly useful. To optimize the lipase manufacturing as well as oil hydrolysis process the Statistical methods have been adopted. *Bacillus* spp. pool isolated from matured contaminated soil of petroleum and *B. stearothermophilus* isolated from slaughter house waste hold for promising bioremediation [446]. Under acidic conditions *Burkholderia* sp. and *Raoultella planticola* bacterial genera having capabilities to degrade edible oil [447]. For the treatment of high strength Oil and Gas wastewater other bacterial consortia have been articulated as potential inoculum involved of *P. aeruginosa, Bacillus* sp., *Halomonas* sp., *Citricoccus alkalitolerans* and *Acinetobacter caloaceticus* revealed in another studies [448–450]. And other one comprised of *B. subtilis, B. licheniformis, B. amyloliquifaciens, S. marsescens, P. aeruginosa*, and *S. aureus* [12].

Lipolytic activities have also been practically applied for bioremediation using fungal culture is *Geotrichum candidum* applied in olive mill wastewater treatment and solid culture of *P. chrysogenum* sapplied in bioremediation of waste cooking oil characterize this statement. In the treatment of waste water *Y. lipolytica* has found massive uses [10, 128, 451]. The Antarctic basidiomycetous yeast *Mrakia* *blollopis* SK-4 has been productive in the low-temperature remediation of milk fat curdle [452]. In Oil and Gas biodegradation symbiosis of Yeast–bacteria is also valuable as shown in the association between lipase-secreting *Burkholderia arboris* and glycerol-assimilating *C. cylindracea* [453]. Quizalofop-*p*-ethyl (QPE; ethyl(R)-2-[4-(6-chloroquinoxalin-2-yloxy) phenoxy] propionate) is a member of the aryloxyphenoxypropionate (AOPP) group of herbicides is a post-emergence effectively controls grass weeds and is often detected in the environment degraded *Pseudomonas* sp. J‑2 was isolated from acclimated activated sludge [454].

#### Lipase in biodegradation of oil

In cold environments the biodegradation of petroleum hydrocarbons such as Alpine soils able to degrade these contaminants is a result of indigenous cold-adapted microorganisms [455]. *P. putida* GPo1 alkB; *Acinetobacter* spp. alkM; *Rhodococcus* spp. alkB1, and *Rhodococcus* spp. alkB2 in the degradation of n-alkanes, *P. putida* xylE in aromatic hydrocarbons, *P. putida* ndoB and *Mycobacterium* sp. strain PYR-1 nidA in the polycyclic aromatic hydrocarbons the seven genotypes determined form the 12 samples taken from the oil-contaminated sites [456, 457]. Bacterial monocultures showed positive response in bio-augmented clean-up of waste water effluent polluted with hydrocarbons and organic polymers using hydrolytic enzymes isolated from lubricant-contaminated effluent from an electric power station [458, 459]. In freshly polluted unfertilized and fertilized soils monitored the activity of microbial lipase is a valuable display of diesel oil biodegradation. In the coastal environment the fungal species can be used to destroy the oil spills which may improve ecorestoration as well as in the enzymatic oil processing in industries [460]. Several microbes such as *B. aliphaticum, Edwardsiella tarda, Bacterium aliphaticum, Bacillus megaterium, Bacillus cereus, Pseudomonas maltiphilia, Fusarium vertiaculloide, Botryodiphodia thiobroma, Fusiarum oxysporum, Cryptococcus neofomas, Aspergillus nige*r and *Candida tropicalis* have been reported for the production of lipase enzymes and have potential to degrade crude oil [10].

#### Lipase in pulp and paper industry

Lignocellulosic biomass in enormous quantities processes in pulp paper industry every year. For the manufacturing of pulp this machinery is highly diverse and several prospects occur for the presentation of microbial enzymes [461]. In the paper industry enzymes have found some uses but these have been confined mainly to areas such as alterations of raw starch. For waste paper deinking lipase can increase the pulping rate of pulp, intensity and whiteness decreases chemical usage, pollution level of waste water, prolong equipment life, conserve energy and time and decrease the composite cost [462]. To a deinking arrangement for ethylene oxide–propylene oxide adduct stearate upgraded whiteness of paper and reduced enduring ink spots using lipase from *Pseudomonas* sp. KWI-56 [463]. The monitoring approach of enzymatic pitch has been in use in a large-scale paper-making process as a routine operation since early 1990s using lipases [464].

#### Lipase in leather degreasing

For removing the fat lipases distinguish a more ecologically sound technique. Lipases permit tensides to be substituted entirely for bovine hides [34, 465]. The use of solvents is very common and these can also be substituted with lipases and surfactants which contain up to 40% fat for sheepskins [466]. For sheepskins if the surfactants are used they may be harmful to the environment and are usually not as effective [467]. Small animal skins and hides from intensively fed cattle the degreasing is a necessary stage in the processing of fatty raw ingredients [468]. Volatile organic compound (VOC) emissions are harmful in environmental concern generated from using organic solvents and surfactants conventional methods. In moderate fat content the fats and grease removes from skins and hides by lipase enzymes [469]. In skin and hide degreasing both alkaline stable and acid active lipases can be used. Triglyceride hydrolysed to glycerol and frees fatty acids using lipases [470]. The degradation of fat cell membranes and sebaceous gland components the alkaline stable proteases are used to encourage and to improve the process. For using lipases deliming and bating are the most suitable processing stages [471, 472]. Acid active lipases have been stored in a pickled state can be used to treat skins. The uniform colour and a cleaner appearance are the main improvement of using lipases [473]. Production of hydrophobic (waterproof) leather upgraded using lipases, leather manufacturers have commented that ‘fogging’ is reduced for car upholstery [334]. Fat dispersion and production of water-resistant and low-fogging leathers are the two advantages over the solvents or surfactant proposed the tanner by lipases. During soaking and/or liming the alkaline lipases are applied in combination with the relevant protease preferred [474]. Between the other possessions, making the fat manageable to the lipase the protease will open up the membranes surrounding the fat cell [475, 476]. The breakdown foodstuffs emulsify the intact fat and the fat becomes more mobile, then which will distribute itself through the covering so that in several cases a suitable degreasing with surfactants will not be compulsory [477]. The lipases (acid) can also be applied for instance pickled skin or wool and fur, or semi acid for wet blue Wool in an acid process [478]. The combination of an acid lipase and an acid protease enzyme the example are Novozyme, Denmark markets NovoCor ABL and NovoCor ADL, NovoLime for acid bating of fur and wool; for enzyme-assisted liming of hides and skins a protease/lipase blend; an acid lipase for degreasing of hides and skins are NovoCor AD [479, 480]. Lime and mixtures of sodium sulphide to dissolve hair present on the skins for the treatment of animal skins has used conventionally in the leather engineering industry but this approach is unpleasant and polluting both [481]. The liming is not efficient in chemical processes where the elimination of remaining fats and protein fragments are allied with the hide and the hair. To utilize a mixture of lipases it has become common practice for this purpose and also known as technical jargon as the bating process [482, 483]. The hair on the skins becomes slackens and the enzymes removes, which can then be clarified off. Using the traditional approaches compared to leather manufacturing the end product is of a higher quality. For the degreasing of suede clothing leathers from wooled sheep skins the lipase was used from *Rhizopus nodosus* [484, 485].

#### Lipase in plastic biodegradation

To curtail the environmental complications widely used of biodegradable plastics as a clean and green technology processes though there are biodestructible plastics are used interchangeably despite their transformations [486, 487]. The extent and rate of degradation is the main difference between the biodestructible and biodegradable plastics, where the former necessitates further management unlike the latter. The comprehensive destructibility of plastics is based on the capability of lipases to cut down polycaprolactone (aliphatic polyester); to promote their rate of degradation can be diversified with plastics observed in Fermentation Research Institute Tsukuba, Japan [488]. Lipase producing species of bacteria are applicable to biodegradation of Polyurethanes (PUR) are *Pseudomonas protegens* BC2-12, *P. protegens* CHA0, *P. protegens* Pf-5, *P. fluorescens* A506 and Pf0-1, *P. chlororaphis* [489]. To act on PUR one of the first enzymes identified was the PueB lipase from *Pseudomonas chlororaphis*. *Pseudomonas* sp. genus of Gram-negative betaproteobacteria has been linked with PUR activities most regularly [490]. At least one additional enzyme active on PUR codes for organisms and labelled as PueA, the secreted hydrolases degraded PUR and the degradation is tightly regulated. From *Pseudomonas pelagia* (PpelaLip) a putative lipase recognized as prospective enzymes performing on polyesters in broad-spectrum using an in silico genome mining approach [491]. Polyurethane was degraded significantly by *Pseudomonas* sp. The production of high amounts of extracellular lipases in *P. aeruginosa* was reported to facilitate the degradation of aromatic–aliphatic polyesters and polyesteramides [492].

#### Lipase in polymer degradation

Lipase isolated from the *Thermomyces laguginosus* (TLL) is a prominently thermostable basophilic enzyme and have capability in both immobilized and soluble form [493, 494]. In the current scenario the most significant ecological complications are the degradation of polymers. Several lipases were performed the catalysis of the side chain of poly (vinyl acetate) in toluene at 60 °C [495, 496]. The hydrolysis of longer side chains are in order hog-pancreas lipase > Novozyme 435 > TLL > *Candida rugosa* lipase whereas in the reverse order the short chains are hydrolyzed. On the biodegradation of poly (-caprolactone) the effect of several solvents is another example [497]. Using two different lipases Novozyme 435 and TLL the reaction was implemented at 45 °C, while with viscosity the rate of degradation reduced and the polarity of the solvents increased [498, 499]. In non-aqueous solvents the inactivation rate was greater using TLL than Novozyme 435. The polymers at an optimal value concentration of 8.7 wt.  % of water in acetone both are the enzymes exhibited the maximum degradation [500]. TLL degraded another polymer was Poly (bisphenol-A carbonate). At various temperatures (26–70 °C) the reaction was executed in solution by altered lipases in various solvents *Candida rugosa*, hog-pancreas, TLL and Novozyme 435 [501]. For hog-pancreas lipase and other lipases the optimal temperatures were 50 and 60 °C. The degradability activity overall of the lipases was TLL > Candida rugosa > Novozyme 435 > hog pancreas order [502]. The viscosity and polarity of the solvents effect was the same as exhibited in the degradation of poly-caprolactone. From *Thermobifida fusca* and *Fusarium solani* TLL and cutinases hydrolyzed poly (ethylene terephthalate) textiles and films and bis (benzoyloxyethyl) terephthalate endo-wise is another example [503]. A seven-fold increase of hydrolysis products released from 3PET was determined in the presence of Triton X-100 due to interfacial activation of the lipase. Semi-crystalline poly (ethylene terephthalate) films and fabrics increased hydrolysis rates were observed for both lipase and cutinase in the presence of the plasticizer *N*, *N*-diethyl- 2-phenylacetamide [502, 503]. Enzymes from *Penicillium citrinum, Thermobifida fusca, Fusarium solani pisi* and TLL treated with the linear aromatic polyester poly (trimethylene terephthalate). The highest amounts of hydrolysis products cutinase from the polymer were found to release from *T. fusca,* and capable to hydrolyse and open a cyclic dimer [504]. So in the degradation of several polymers that pretense an actual ecological complication TLL was found very applicable [505, 506].

#### Lipase in detergents manufacturing

The chemical constituents of detergents caused ecological contamination and to hazardous for the fauna and flora so the lipases are used as a substitute of these unsafe constituents [507, 508]. From the muddy substrates the lipid molecules removed lipase based detergents which preferred for long life of cleaned fabric and active at the ambient temperature [509, 510]. So, currently most of the industries producing enzymatically based detergents. *Pseudomonas* ADT3 produced lipase was found valuable in detergent [511]. The removal of corn oil stains from un-dyed cotton fabric takes place when lipase mixed with the detergent and extracted from *Bacillus sonorensis* [512, 513]. At low temperature to wash the cloths in laundry the cold active lipases are useful as additives in detergent preparations [514] and in organic mixture of chiral intermediary. *Pseudomonas aeruginosa* strain BUP2 produced an alkaline and thermotolerant lipase used in the detergent industry efficiently with high specific activity [515]. Commonly used in detergents the lipase producing microbes are *Bacillus flexus* XJU-1, *Bacillus licheniformis*, *Bacillus licheniformis* VSG1, *Bacillus pumilus* SG2, *Bacillus subtilis* JPBW-9, *Geobacillus* sp., *Pseudomonas aeruginosa* sanai and *Serratia marcescens* DEPTK2 [516, 517]. *P. mendocina* (Lumafast) and *Pseudomonas glumae* bacteria produced lipases with high temperature optima for commercial detergent formulations are used [518]. *P. mendocina* and *Pseudomonas alcaligenes* produced lipases known as Lumafast and Lipomax, respectively, by Genencor International, AU-KBC Research Center, Life Sciences, Anna University, Chennai, India (http://www.au-kbc.org/beta/ bioproj2/uses.htm) [519–521]. An alkaline lipase produced bacteria *P. alcaligenes* M-1, have capability to removing the fatty stains using in washing machine [522]. Solvay Enzyme Products, Inc. 1992-01-29/1990-07-25, extracted from *Pseudomonas plantarii* is a nonionic and/or anionic detergent formulation patented in a European Patent Office (EPO) [523]. Lipolase introduced by Novo Nordisk In 1994 was the first commercial lipase which was extracted from *Trichoderma lanuginosus* and expressed in *A. oryzae* [524, 525]. Lipo Prime^®^ is a lipase containing detergent also produced by them. *Trichosporon asahii* MSR 54 produced an alkaline lipase and developed a presoak formulation which applicable for the removal of oil stains at ambient temperature [526].

#### Lipase in resolution of racemic mixtures formation

To resolve the racemic mixtures and to synthesize the chiral building blocks lipases can be used for pharmaceuticals, agrochemicals and pesticides [527]. In nonpolar organic solvents some lipases retains their movement [528, 529]. In the hydrolysis of water-insoluble esters they can be used through stereospecific hydrolysis in the resolution of racemic mixtures [530]. Enantio selective hydrolysis or esterification, have been developed the resolution of stereoisomers [468, 531]. In the efficacy of many drugs chirality is a key factor; so in the pharmaceutical chemistry the production of single enantiomers of drug intermediates has become increasingly [532, 533]. For the preparation of bulk drug ingredients and agricultural products the chiral intermediates and fine chemicals are in great claim from the pharmacological and agrochemical manufacturing [534]. The vast prospective of microorganisms there has been an increasing wakefulness and enzymes for the conversion of artificial compounds with more chemo-, regio- and enantioselectivity [535]. The process of enantioselectivity esterification with alcohols lipase from *C. antarctica* (Novozyme (R) 435) has been used for the kinetic resolution of racemic flurbiprofen [536, 537]. RS-beta-(aminomethyl)-4-chlorobenzene propanoic acid applied in the remedy of pain and as a muscle relaxant chemically known as baclofen and produces two isomers. For resolving racemic mixture lipase extracted from *C. cylindracea* has been used as a catalyst [538]. Stereoselective acetylation of racemic 7-[*N*, *N*’-bis-(benzyloxy-carbonyl) *N*-(guanidinoheptanoyl)]-alphahydroxy-glycine 24 to corresponding S-(−)-acetate 25 was demonstrated catalysed by lipase [539]. For the total chemical synthesis of (−) -15-deoxyspergualin 23 an immunosuppressive agent and antitumor antibiotic and S- (−)-acetate 25 is a key intermediate [540, 541].

To prepare chiral intermediates for pharmaceuticals biocatalytic processes were used and includes the subsequent methods [542, 543]. A key chiral intermediate is (S) [1-(acetoxyl)-4-(3-phenyl) butyl] phosphonic acid diethyl ester 21 and essential for total compound mixture of BMS-188494 (an anticholesterol drug) [544]. Stereoselective acetylation of racemic [1- (hydroxy)-4-(3-phenyl) butyl] phosphonic acid diethyl ester 22 using *G. candidum* lipase prepared BMS-188494 (an anticholesterol drug) using a chiral intermediate [545, 546]. Lipase B from *C. antarctica* was revealed the enzymatic determination of racemic 2-pentanol and 2-heptanol [547, 548]. For the production of anti-Alzheimer’s drug required a chiral intermediate known as S- (+) - 2-pentanol. Under a license from the Massachusetts Institute of Technology a company Chemie Linz Co. (Austria) manufactured phenoxypropionate herbicides is being carried out on a 100-kg scale by the resolution of 2-halopropionic acids [549]. For the manufacturing of optically active intermediates on a kilo-gramme scale several pharmaceutical companies used lipases world-wide [334]. In the UK, Enzymatix companies offer a whole variety of intermediates prepared via lipase mediated resolution specialize in biotransformation [58, 550]. Polyfunctional organic compounds of regioselective modifications are another area of intensifying lipase solicitation [551]. Castanospermine is a favorable drug for the curing of AIDS magnificently prepared by using lipase in regioselective modification [552].

### Applications of lipase in pharmaceuticals and medical industry

#### Lipase in pharmaceuticals

A thermo-stable lipase has capabilities to catalyzing in bioenergy, pharmaceutical manufacturing and for trans-esterification of palm oil to FAMEs also resistant to organic solvents obtained from *Acinetobacter baylyi* [553, 554]. Furthermore, lipases are also applicable for the curing of hair loss and skin scalp disease [555]. For the industrial production of aryl aliphatic glycolipids, citronellol laurate from citronellol and lauric acid, and ethyl esterification of docosahexaenoic acid to ethyl docosahexaenoate the cold active lipases are used [556]. A very less quantity of enzymes exhibited positional specificity but *Bacillus* lipases showed selectivity to the fatty acid chain length of an ester [557]. In pharmaceutical industries for the synthesis of enantiopure compounds *Bacillus* lipases can be used due to these properties [558]. Using *Staphylococcus* lipase the antioxidant properties such as tyrosol acetate, propyl gallate and eugenol benzoate are manufactured [559]. In the case of tuberculosis (TB) detection lipase can be used for the diagnostic purposes. *Mycobacterium tuberculosis* lipase is used to check the infection with high specificity and sensitivity detection [560].

In blood serum the level of lipase for the detection of acute pancreatitis and their wound the level of lipase can be used. Due to using the overdose of alcohol or bile duct obstruction caused pancreatitis [561, 562]. As a constituent of topical anti-obese creams lipases are used in manufacturing of hair waving and also used for the curing of malignant tumors as digestive aids because lipases are initiate as activators of tumor necrosis factor (TNF) [131, 563]. Lovastatin drug reduce the serum cholesterol level and manufactured from *Candida rugosa* lipase [564]. The diltiazem hydrochloride is a widely used for the vasodilation of coronary and manufactured from *S. marcescens* lipase using a key intermediate 3-phenylglycidic acid ester by asymmetric hydrolysis [565]. For the manufacturing of (2R, 3S)-3-(4-methoxyphenyl) methyl glycidate (a key intermediate for diltiazem) and 3, 4-dihydroxylphenyl alanine (DOPA, for curing of Parkinson’s disease) microbial lipases (EC 1.10.3.2) are used [566]. Lipases (EC 3.1.1.3) enzymes are used in the organic synthesis and also for optically active alcohols, acids, esters, and lactones [567].

#### Lipase CalB for Odanacatib

**Fig. 8:**
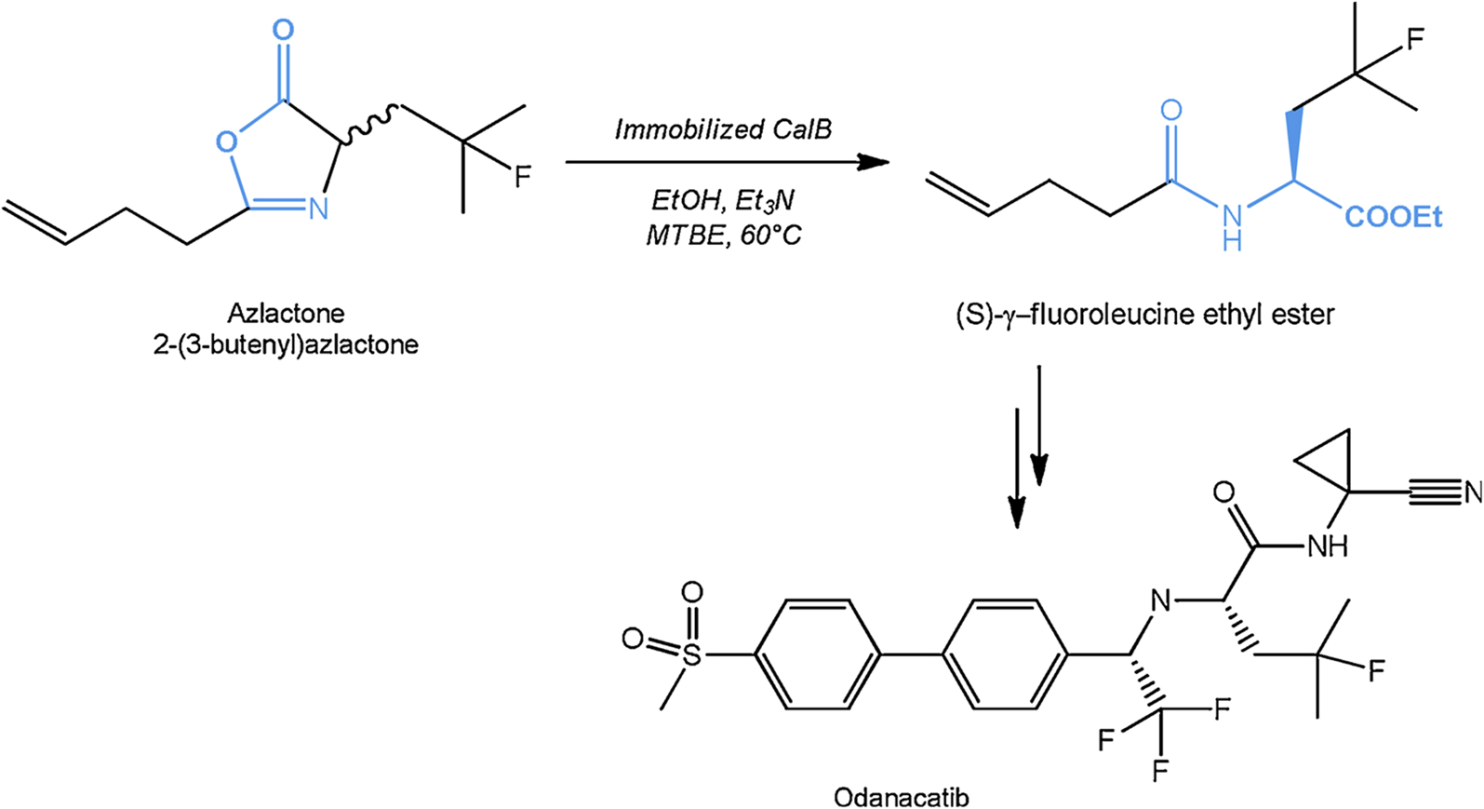
Lactone opening and esterification by immobilized CalB in organic solvent to produce chiral intermediate of drug Odanacatib

Generally, Lipase (EC 3.1.1.3) isolated from *Candida antarctica* (CalB) are commonly used as catalyst for the manufacturing of personal care products, active pharmacological and food constituents due to their regio-, chemo- and enantioselectivity [568]. Due to its broad selectivity and high acceptance to organic solvents and temperature CalB finds several uses in industrialized procedures in immobilized form [569, 570]. Merck introduced in 2011, using immobilized CalB for the manufacturing of Odanacatib. Odanacatib discovered in 2008, is a potent cathepsin K inhibitor and was estimated for the curing of osteoporosis in women after menopause and at present it is withdrawn [571, 572]. The ethanolysis of azlactone is the complete ring opening vital step the procedure reported by Merck [573]. Using the high substrate concentration (200 g/l of azlactone) in a continuous plug flow reactor at 60 °C in methyl tert-butyl ether (MTBE), after reaction catalyzed the immobilized CalB gives 95% conversion of the desired (S)-γ-fluoroleucine ethyl ester [574, 575], (Fig. 8).

For the process due to the key success the usage of several carriers than the one that is normally working for CalB immobilization [576], which allowed 99.9% decline in price when matched to expending the industrially obtainable Novozym 435 (CalB immobilized on a divinyl-benzene/methacrylate carrier) [577, 578]. The stability and movement was improved expressively when immobilizing CalB on an octadecyl functionalized methacrylate resin equated to Novozym 435 preparation which is commercially available [579]. To the optimal interaction of the hydrophobic octadecyl groups with lipases this was renowned. In the immobilization of transaminase the octadecyl functionalized carrier also shows good performance [284, 580].

#### Lipase CalB for Sofosbuvir

A chronic liver disease Hepatitis C is an infectious disease affected by Hepatitis C virus (HCV), within the Flaviviridae family and a member of the hepacivirus genera worldwide [581]. It is an asymptomatic infection which traumatized to liver and finally, to cirrhosis and the symptom apparent after many years generally [582, 583]. The cirrhosis of liver exhibited into liver failure oesophageal, gastric varices and finally cancer. The direct contact with infected blood the HCV is predominantly transferred [584, 585]. The high mutagenicity of HCV and the existence of several genotypes and subtypes is the result of emerging upgraded approaches of treating hepatitis C [586].

Several chemical steps are required for the production of Sofosbuvir which is an enormous compound, for the enantioselective hydrolysis of an acetate ester into the chiral alcohol into the procedure immobilized CalB is used in the patent from Gilead [587]. Divinyl benzene/methacrylate polymer (Novozym 435) was used in MTBE on CalB immobilization with aqueous 0.1 M phosphate buffer pH 7 soaking at a temperature of 10 °C and the racemate conversion to the favorite enantiomer is almost 40% [588]. An intermediate manufacturing of sofosbuvir another biocatalytic process was reported in 2014 by Chemelectiva-HC-Pharma [311]. The use of immobilized lipase CalB the process also involves but the catalyzed reaction excitingly is on a diverse position of the molecule [589, 590]. Indeed, in polar protic organic solvent to give the corresponding alcohol at 60 °C the immobilized CalB is used for a regioselective mono-deacetylation of sofosbuvir intermediate.

#### Lipase in diagnostic tool

In the medical sector lipases are significant drug targets or marker enzymes. Their presence or increasing levels can indicate certain infection or disease and can be used as diagnostic tools [591]. To generate glycerol lipases are used in the enzymatic determination of serum triglycerides and consequently analysed using the enzyme interconnected colorimetric reactions [592]. The acute pancreatitis and pancreatic injury detection the level of lipases in blood serum can be used as a diagnostic tool [593]. The serum amylase and lipase levels are used to confirm the analysis of acute pancreatitis but the ultrasonography, computed tomography and endoscopic retrograde cholangiopancreatography are the most accurate laboratory indicators for pancreatitis at serum trypsin level [594]. Using lipases some new improvements in the diagnosing pancreatitis have been produced. Serum lipase activity is not specific for pancreatitis or exocrine pancreatic insufficiency (EPI) because several cell types secrete lipases [595]. For exocrine pancreatic function the concentration of serum pancreatic lipase immunoreactivity (PLI) is highly specific and sensitive for pancreatitis [596]. A newly developed Feline Pancreatic Lipase Immunoreactivity (FPLI) serum test and primary results recommend that for the diagnosis of feline pancreatitis this test is more sensitive comparison to another diagnostic tool [597]. A wide variety of virulence-related genes are found in drinking water possess of *Aeromonas* bacteria that recommends the examining importance of several isolates as possible in order to improved appreciate the health hazard present in bacteria [598]. In municipally treated drinking water the indication of *Aeromonas* bacteria represents the potentially pathogenic *Aeromonas* bacteria on the bases of virulence factor characterization. *Propionibacterium acnes* lipase examined for the skin diseases and Unsei-in [599]. In axillary seborrheic dermatitis (ASD) the butyric acid production was more than in other dermatitis, and that in acne vulgaris (AV) was more comparison to controls [600]. In acne vulgaris (AV) the *Propionibacterium acnes* lipase is the pathogenic factor and in ASD the fatty acid production by lipase may be pathogenic factor [601]. An opportunistic pathogen *P. aeruginosa* have capabilities to produce and secrete several virulence factors contributing to the pathogenicity of *P. aeruginosa* regarded as biological properties [602]. The pathogenic bacteria like *P. acnes*, *Corynebacterium acnes* and *Staphylococcus aureus* lipases has also been initiate to have the influence on skin rash in acne patients [603].

#### Lipoprotein lipase (LPL) activators as anti-obesity drugs

A number of physiological procedures containing homeostasis also secrete an adipocytokine called LPL controlled by adipose tissues [604]. The fatty acids separating (triglycerides derivative) modified by adipose tissues among the metabolism of plasma cholesterol, several tissues and successive intracellular procedures related and dependent on accessibility of lipid [605]. TAGs are deposited in adipose tissues and skeletal muscles are produced by adipocytes. Between the muscle and adipose tissues the partitioning of plasma TG disrupted due to the inequities in LPL activity and their availability, this may also lead to and obesity and insulin resistance [606, 607]. An enhancement in LPL activity has been regularly recognized in obese individuals [608]. The effect of LPL activity and fat deposition correlated in the transgenic mice which were carried out for examinations [609]. To controls in adipose and heart tissues and smooth muscles of transgenic mice the LPL activities were found to be higher compared to control mice observation [610]. Though, there was no alteration in fat accumulation quantity of which showed that obesity was not brought due to advanced LPL activity [611]. The triacylglycerols from lipid droplets of adipocytes hydrolysed by the hormone sensitive lipases (HSL) and advised the elevation in transgenic mice [612]. In the modulating of overall weight gain the HSL is dominant directed by it. So, it may advise that the physiological modulation of LPL activity can be utilized for the control or the causes of the metabolic disorders [613]. As contrasting to adipose tissues the LPL execute the oxidation of fat in skeletal muscles mainly. In the skeletal muscles of transgenic mice hinders diet-induced obesity [271]. Diet encouraged over-expression of human LPL hinders the diet-induced obesity in the skeletal muscles of transgenic mice [614, 615]. The ratio of carbohydrate: fat oxidation remains unperturbed indicated in the observation of constant Respiratory Quotient (RQ; moles of CO_2_ production per mole of Oxygen consumed) [616]. A relationship between the RQ and body weight revealed in previously published analysis. In smooth muscles of Pima Indians the RQ is inversely relative to LPL activity [617, 618]. LPL activator NO-1886 reduces RQ with long-term treatment and decreases accumulated fats fed with elevated levels of fructose in diabetic rats [619]. For lipid- and non-lipid-associated obesity as a therapy associated to non-specific anti-obesity drugs the specific LPL activators may substantiate much valuable with respect to skeletal muscle or tissues [620, 621].

#### Potential use of lipases in treatment cancer

Due to reducing physical activity in routine life and in taking high calories may responsible for the risk of liver, colon breast, pancreas, and prostate cancers [622, 623]. Consequently, the intensities of triglycerides (TG) in the serum displayed and may be influenced the colorectal and pancreatic cancers or precancerous lesions. The hydrolysis of plasma TG catalyzed by the lipoprotein lipases (LPL) is also recognized [624]. It is predicted that in human the short arm of chromosome 8 which bear a putative tumor suppressor gene deleted to initiate or promote hepatocellular carcinoma is reported. Using the FISH analysis it is proved in evidence the LPL- deficiency promotes the prostate cancer [329, 625]. Other cancer susceptible genes also deleted with the short arm of human chromosome which is responsible for breast cancer 2(DBC2), liver cancer 1 (DLC1), mitochondrial tumor suppressor1 (MTUS1) [626]. Therefore, on this chromosome the LPL gene deletion moves the proximal cancer related genes in interrogation and their joint effect in the promotion of carcinogenesis are reported [627]. Patients suffer a loss of skeletal muscle and adipose tissues associated with cachexia (weakness and wasting of the body due to severe chronic illness) are the several forms of cancer, the lipid metabolism and triglyceride hydrolysis is associated with cachexia [628, 629]. LPL acts on monoglycerides (MG) and triglycerides (TG) which shows a key role in lipids and lipoprotein metabolism. LPL modulators such as tumour necrosis factor (TNF)-α, Interleukins (IL-1, IL-6) induce by cachexia which obstructs the activity of LPL foremost to a stark cut in the accumulation of fatty tissues [628]. To progress the LPL activity to control the cachexia in cancer patients these outcomes overlay the approach for further research [630].

#### Lipases in medical devices

Docosahexaenoic acid (DHA) and eicosapentaenoic acid (EPA) fatty acids (FA) are significant in growth and development, fat malabsorption can lead to decreased caloric intake deficiencies of fatty acids (FA) and caused cystic fibrosis and exocrine pancreatic insufficiency [631]. Cystic fibrosis patient used pancreatic enzyme replacement therapy in conjunction with meals to increase the absorption of fat and other nutrients [632]. The enteral nutrition allows them to meet growth and weight goals thousands of cystic fibrosis patients used in USA [633]. Due to the poor stability of hydrolyzed fats the formulas available contain triglycerides rather than fatty acids. Alcresta Therapeutics developed a single-use cylindrical cartridge in collaboration with Chiral Vision with a closed chamber by frits comprising digestive immobilized enzyme that links in-line with the enteral feeding set [634]. On a methacrylic polymer (EC 3.1.1.3) the cartridge contains covalently immobilized lipase isolated from *Chromobacterium viscosum*, *Pseudomonas fluorescens, Burkholderia cepacia*, or *Rhizopus oryzae* on polymeric beads that hydrolyses up to 90% of the fats passed through the cartridge in the enteral formula [385, 635]. Since the device increases the life prospect of the patients and help to fat absorption augmentation consequently improving chronic lung disease and cognitive ability with a reduction time for parenteral nutrition [636].

### Application of lipase in bioenergy/biodiesel production

Due to increasing the environmental pollution issues such as climate changes, greenhouse gases and increasing the prices of fossil fuels have encouraged the examination into the improvement of biofuel/biodiesel technology from sustainable resources [637]. So, the application of lipolytic enzymes not only helps to alleviation the enormous amount of lipid waste substances in a sustainable and ecofriendly way but also challenges the energy safety matters and which could substitute for fossil fuels [638]. Due to the outstanding bio physiochemical features of lipases (triacylglycerol acylhydrolases, EC 3.1.1.3) revealed very substantial biocatalysts. The attention is increased due to biocatalysts on the bases of biotechnological applications [639]. The shortest generation time for lipase enzyme production generally the microorganisms are preferred. Other benefits of microorganisms are high productivity of substrate conversion into product, environmental conditions versatility, and simplicity of genetic operation and in harvesting situations [640]. Lipases have capabilities to catalyze the same reaction using from different sources, but the microbial lipases are mostly used for biodiesel production like *Aspergillus niger*, *Candida antarctica, Candida rugosa*, *Chromobacterium viscosum*, *Mucor miehei*, *Lactobacillus plantarum, Pseudomonas cepacia*, *Pseudomonas fluorescens*, *Photobacterium lipolyticum*, *Rhizopus oryzae*, *Streptomyces* sp., and *Thermomyces lanuginose, Burkholderia cepacia, Bacillus subtilis Q1 KX712301* [293, 641]. *Candida rugosa* yeast is mostly used for lipase production. Currently, *Streptomyces* sp. was explored as an effective lipase generating microbe for biodiesel manufacturing and found appropriate in the field of biodiesel [642]. The cost of biodiesel production greatly reduces using waste and non-edible vegetable oil, and measured a significant step in decreasing environmental pollution and recycling waste oil [643]. The short-chain alcohol tolerant abilities of lipase and higher thermostability form very suitable for usage in the production of biodiesel. *Candida antarctica* lipase in immobilized form catalyzed methanolysis of soybean oil for the production of biodiesel [644, 645].

Currently, over the other lipases in terms of energy saving the cold-active/adapted lipases have been found to be attractive for the production of biodiesel, subsequently the synthesis of biodiesel by other lipases was applied at elevated temperatures [325, 370]. Mostly the cold-active/adapted lipases characterized and identified are bacteria and only few fungal isolates have been also reported are *Aspergillus nidulans* [214], *Geotrichum* sp. [646], and *Penicillium expansum*. Immobilized lipase from *Pseudomonas fluorescens* is the most dynamic biocatalyst, followed by *Pseudomonas cepacia* immobilized lipase [647]. *Aspergillus awamori* BTMFW032 a marine fungus isolated from seawater was detected to harvest an extracellular lipase and biodiesel production [648]. For biodiesel production only microbial lipases are the material of practical importance, because these are produced in industrial scale [649, 650].

The greatest extensively used in biodiesel production are free lipases between the commercially available lipases from *P. fluorescens* (Lipase AsK, Amano), *B. cepacia* (Lipase PS, Amano), and *T. lanuginosus* (Lipase LA201 and Lipopan 50BG, Novozymes), and immobilized lipases from *T. lanuginosus* (Lipozyme TL IM, Novozymes) and *R. miehei* (Lipozyme RM IM, Novozymes) [651, 652].

In *A. niger* CALB expressed and immobilized onto an acrylic macroporous resin between recombinant lipases that is known as Novozym 435 (Novozymes) commercially lipase widely used for the production of biodiesel [77, 303]. In conventional biodiesel production this recombinant lipase has been successfully used, as well as using isopropanolysis of soybean oil in biodiesel production, the synthesis of biodiesel and glycerol carbonate simultaneously from corn oil as the acyl acceptor using dimethyl carbonate, and production of so-called “Ecodiesel” [353, 653].

### Application lipase in textile industry

For degreasing the textile raw materials and increasing the performance lipases are mainly used in the textile industry [654]. The studies of physical and chemical changes of the treated wool fiber and the commercialization of lipase have been observed [655]. On the surface of wool fiber the fatty acids are found discarded treated with anhydrous alkaline lipase and also augmented the quality of wool [656]. The dewaxing effect of silk fibers with lipase and dewaxing and degumming on silk fiber simultaneously the effects of lipase and protease with proper uses and doses have been assessed the better qualities of fiber such as rate of weight loss [657], dyeing, wettability, microstructure, gloss and other properties comparison to without uses of lipases [658]. Additionally, the desizing process of cotton fabric, amylase and lipase can also be decreasing the degree of pollution of the wastewater and degrading the starch into water-soluble compounds [659].

### Safety evaluation of lipases

In agreement with the provisions and conditions of use provided for in Article 7(2) of Regulation (EC) No 1332/2008 on food enzymes [660]. For the valuation of safety and the consent process of food additives, food enzymes and food flavourings Regulation (EC) No. 1331/2008, recognized the European Union (EU) procedures [661]. In Union list only food enzymes involved may be placed on the market as such and used in foods [578]. Microbial lipases used in food applications do not display any toxicity so it is significant in nature. Testing involves for the evaluation of safety on the bases of acute, sub-acute and sub chronic oral toxicity and mutagenic potential [662]. Lipase G produced from *P. camembertii* was categorized as a nonpathogenic and as nontoxic for the enzyme production employee, operators and the consumer, which is used in the food industry as a processing aid [663]. Under organized fermentation environments lipase derived from *R. oryzae* used as a food additive and the toxic assessment identified for safety concern [664, 665]. *P. pastoris* used in the manufacture of food enzymes preparation also fulfill accepted safety criteria for the use in the degumming of edible vegetable oil against BD16449 phospholipase C. *R. miehei* lipase at high levels expressed in *A. oryzae* exhibited significant effects upon body weight and energy metabolism [484]. From *R. oryzae* Lipase D used for interesterification of edible fats and oils and selective hydrolysis of triglycerides, no adverse effects have been seen when used as designated in the processing of dietary fatty acids and glycerides of fatty acid [127]. *C. rugosa* lipase enzymes engaged in the production of flavours are considered as safe to workers and consumers [141, 666]. *Trichoderma reesei* RF10625 is a genetically modified strain produced triacylglycerol acylhydrolase (EC 3.1.1.3) food enzyme used in baking and cereal‐based processes [13, 578]. The enzyme is free from viable cells of the production organism and recombinant DNA and genetic alterations do not give rise to safety concerns [577, 667, 668].

## Conclusions and future perspective

For lipolytic enzymes lipids and other compounds suitable as substrates through the food processing released into the environment, due to the obstruction edible oil, dairy industry thus creating problems in the biochemical processes and decrease the activity of biomass due to commencement. Microorganisms have capabilities to biodegrade the lipid waste in mild conditions efficiently producing lipolytic enzymes compared to the classical lipid degradation processes leading to environmental sustainability. The hydrolysis of ester bond-containing synthetic plastic, pesticide, insecticide and parabens are the one emerging aspect in current scenario and also applied for the production of bioenergy and energy saving to sustain the global hazardous wastes. Another important aspect is the production of high value-added products using less energy consuming enzymatic catalysis connected with microbial lipases. For the designing of therapeutic and diagnostic aids lipases have become broader and are evolving rapidly as prime candidates currently. For the pharmaceutical and medicinal applications lipase enzymes used as modulators such as activators and inhibitors specifically for handling of lifestyle diseases such as obesity. In the present time modulators have a huge impact on therapeutics and would be further augmented in the imminent future. So the using of these lipases prominently enhances many various biotechnology-based productions.

## Data Availability

The datasets used and/or analyzed during the preparation of manuscript are available from the corresponding author on reasonable request.

## Abbreviations

AV: Acne vulgaris
CALB: *Candida antarctica* lipase B
CRL: *Candida rugosa* lipase
CLA: Conjugated linoleic acid
CLEA: Cross linked enzyme aggregates
CLE: Cross-linked enzyme
CLEC: Cross-linked enzyme Crystals
CSDE: Cross-linked spraydrying enzyme
CTC: Cut-Tear-Curl
DHA: Docosahexaenoic acid
EMC: Enzyme-modified cheese
EMDI: Enzyme-modified dairy ingredients
GRAS: Generally Recognized As Safe
HCV: Hepatitis C virus
HSL: Hormone sensitive lipases
HMF: Human milk fat
HMFS: Human Milk Fat Substitutes
LPL: Lipoprotein lipase
PL: Pancreatic lipase
PLI: Pancreatic lipase immunoreactivity
PUFAs: Polyunsaturated fatty acids
PUR: Polyurethanes
TNF: Tumor necrosis factor
VOC: Volatile organic compound
